# Supplementary figures and images for: Renoprotective effects of paramylon, a β-1,3-D-Glucan isolated from Euglena gracilis Z in a rodent model of chronic kidney disease
Source: PLoS One. 2020 Aug 7;15(8):e0237086. doi: 10.1371/journal.pone.0237086 (PMC7413521; doi:10.1371/journal.pone.0237086)

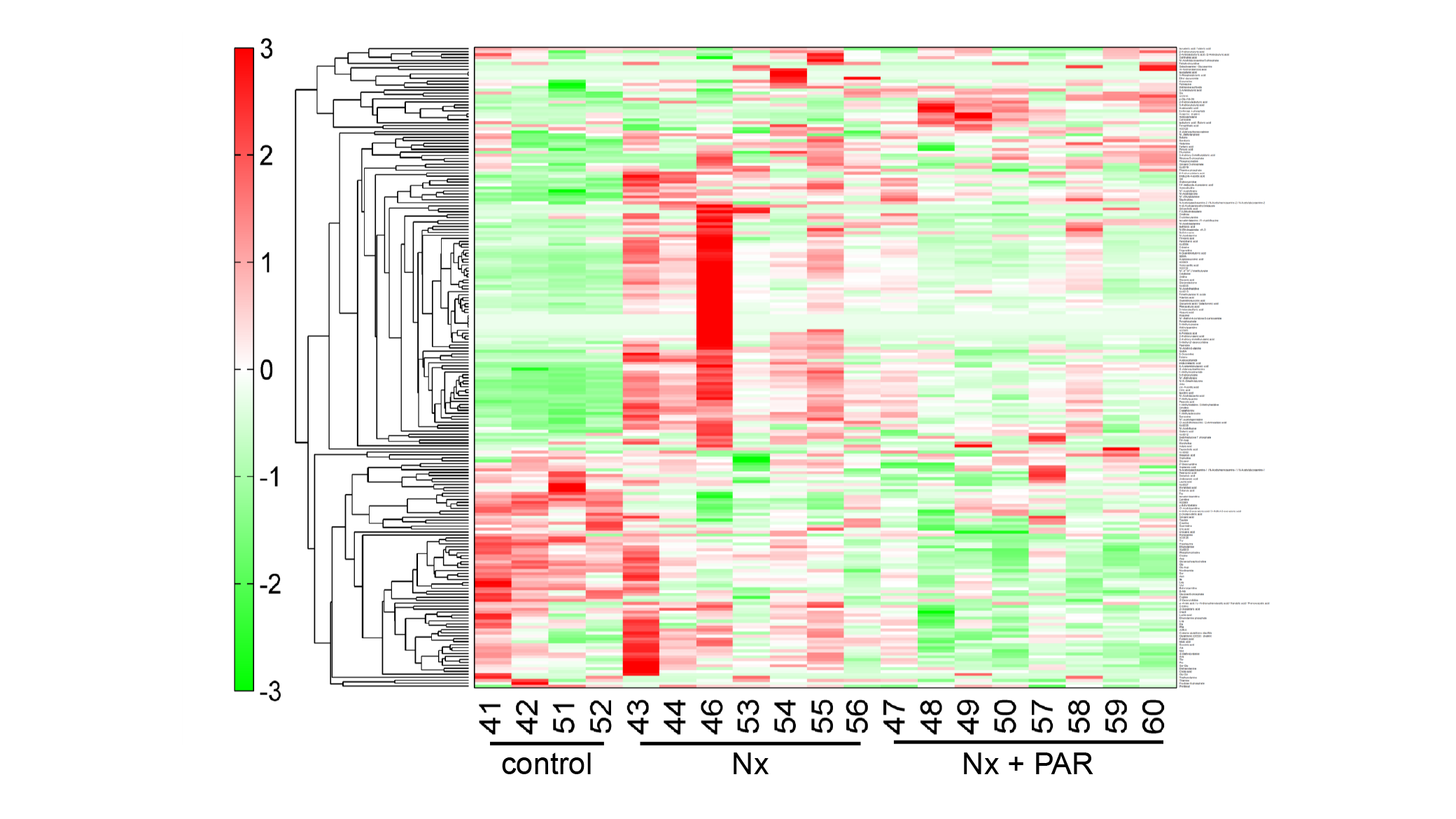

Supplement: S1 Fig — Red indicates higher than average metabolite concentrations, while green indicates those below average. Nx, 5/6 nephrectomy group; Nx + PAR, 5/6 nephrectomy + 5% paramylon treatment group. (TIF) [file pone.0237086.s001.tif]
