# Supplementary material for: Renoprotective effects of paramylon, a β-1,3-D-Glucan isolated from Euglena gracilis Z in a rodent model of chronic kidney disease
Source: PLoS One. 2020 Aug 7;15(8):e0237086. doi: 10.1371/journal.pone.0237086 (PMC7413521; doi:10.1371/journal.pone.0237086)
Supplement: S1 Table — (DOCX) [file pone.0237086.s002.docx]

| Primary antibody | Specification, Supplier | Host species, Type | Dilution |
| --- | --- | --- | --- |
| α-SMA | clone1A4,  Nichirei, Tokyo, Japan | Mouse, mc | Ready to use |
| monocytes/macrophages | clone ED1,  Millipore, Temecula, CA | Mouse, mc | 1:100 |
| CD3 | Abcam, Cambridge, UK | Rabbit, pc | 1:400 |
| PCNA | clone 5A10,  Medical & Biological Laboratories, Nagoya, Japan | Mouse, mc | 1:1000 |

α-SMA, α-smooth muscle actin; mc, monoclonal; pc, polyclonal; PCNA, proliferating cell nuclear antigen.
