# Supplementary material for: Renoprotective effects of paramylon, a β-1,3-D-Glucan isolated from Euglena gracilis Z in a rodent model of chronic kidney disease
Source: PLoS One. 2020 Aug 7;15(8):e0237086. doi: 10.1371/journal.pone.0237086 (PMC7413521; doi:10.1371/journal.pone.0237086)
Supplement: S2 Table — (DOCX) [file pone.0237086.s003.docx]

| Gene symbol | Gene name | Assay ID |
| --- | --- | --- |
| *Ccl2* | chemokine (C-C motif) ligand 2 | Rn00580555_m1 |
| *Tnfa* | tumor necrosis factor alfa | Rn99999017_m1 |
| *Serpine 1* | serpin peptidase inhibitor,  clade E (nexin, plasminogen activator inhibitor type 1), member 1 | Rn01481341_m1 |
| *Il1b* | interleukin 1 beta | Rn00580432_m1 |
| *Tgfb1* | transforming growth factor beta 1 | Rn00572010_m1 |
| *Col1a1* | collagen type I alpha 1 | Rn01463848_m1 |
| *Mmp9* | matrix metallopeptidase 9 | Rn00579162_m1 |
| *Il10* | interleukin 10 | Rn00563409_m1 |
| *Nos2* | nitric oxide synthase 2, inducible | Rn00561646_m1 |
| *Ifng* | interferon gamma | Rn00594078_m1 |
| *Il4* | interleukin 4 | Rn01456866_m1 |
| *Gapdh* | glyceraldehyde-3-phosphate dehydrogenase | Rn01775763_g1 |
