# Supplementary material for: Renoprotective effects of paramylon, a β-1,3-D-Glucan isolated from Euglena gracilis Z in a rodent model of chronic kidney disease
Source: PLoS One. 2020 Aug 7;15(8):e0237086. doi: 10.1371/journal.pone.0237086 (PMC7413521; doi:10.1371/journal.pone.0237086)
Supplement: S4 Table — (DOCX) [file pone.0237086.s005.docx]

| Sample | C1 | C2 | N3 | N4 | N5 | N6 | P7 | P8 | P9 | P10 | C11 | C12 | N13 | N14 | N15 | N16 | P17 | P18 | P19 | P20 |
| --- | --- | --- | --- | --- | --- | --- | --- | --- | --- | --- | --- | --- | --- | --- | --- | --- | --- | --- | --- | --- |
| Rejected hit | 8801 | 10859 | 9176 | 9078 | 11351 | 5708 | 11373 | 13429 | 28200 | 21985 | 18246 | 13302 | 16188 | 15736 | 7250 | 37816 | 25784 | 16648 | 9460 | 22631 |
| Lactobacillales | 18709 | 10649 | 14224 | 12298 | 16235 | 11747 | 14290 | 5539 | 7521 | 5476 | 16644 | 27250 | 12926 | 17767 | 15164 | 2421 | 14542 | 15867 | 12521 | 16986 |
| Clostridiales | 4122 | 1591 | 731 | 2819 | 1154 | 9013 | 5252 | 4554 | 9399 | 6631 | 2030 | 426 | 8378 | 6354 | 25996 | 6262 | 4257 | 5750 | 5934 | 7649 |
| Bifidobacteriales | 1381 | 50 | 2427 | 1247 | 354 | 4711 | 487 | 267 | 290 | 1403 | 22 | 296 | 197 | 3012 | 1400 | 3948 | 85 | 390 | 1440 | 86 |
| Verrucomicrobiales | 69 | 4 | 438 | 308 | 51 | 274 | 180 | 3492 | 809 | 149 | 544 | 0 | 760 | 268 | 189 | 1036 | 307 | 84 | 625 | 704 |
| Pseudomonadales | 18 | 19 | 1 | 6153 | 312 | 90 | 194 | 0 | 0 | 0 | 0 | 0 | 0 | 0 | 2 | 11 | 0 | 0 | 0 | 1 |
| Eggerthellales | 175 | 223 | 115 | 72 | 112 | 76 | 252 | 224 | 436 | 287 | 279 | 106 | 263 | 167 | 429 | 1531 | 177 | 150 | 169 | 335 |
| Bacteroidales | 105 | 190 | 196 | 62 | 121 | 18 | 167 | 66 | 237 | 212 | 408 | 465 | 443 | 212 | 173 | 1651 | 79 | 91 | 86 | 163 |
| Erysipelotrichales | 4 | 1 | 42 | 61 | 20 | 101 | 111 | 67 | 96 | 70 | 2 | 3 | 46 | 167 | 107 | 261 | 108 | 119 | 50 | 135 |
| Micrococcales | 35 | 44 | 7 | 26 | 42 | 9 | 28 | 45 | 162 | 101 | 158 | 85 | 38 | 20 | 37 | 126 | 37 | 20 | 2 | 13 |
| Coriobacteriales | 3 | 2 | 9 | 23 | 8 | 4 | 39 | 53 | 37 | 41 | 26 | 16 | 22 | 10 | 12 | 40 | 34 | 45 | 43 | 69 |
| Corynebacteriales | 3 | 13 | 2 | 108 | 7 | 0 | 1 | 2 | 34 | 0 | 34 | 14 | 9 | 6 | 3 | 34 | 3 | 4 | 4 | 12 |
| Bacillales | 6 | 39 | 3 | 19 | 21 | 0 | 1 | 2 | 70 | 4 | 4 | 2 | 3 | 3 | 4 | 22 | 4 | 0 | 0 | 2 |
| Enterobacterales | 5 | 11 | 6 | 22 | 3 | 0 | 16 | 3 | 2 | 3 | 4 | 7 | 0 | 2 | 8 | 34 | 0 | 1 | 8 | 3 |
| Desulfovibrionales | 10 | 15 | 9 | 0 | 1 | 0 | 1 | 0 | 0 | 12 | 8 | 12 | 17 | 0 | 19 | 12 | 0 | 0 | 2 | 1 |
| Deferribacterales | 0 | 0 | 1 | 4 | 9 | 1 | 2 | 2 | 2 | 4 | 3 | 4 | 29 | 2 | 3 | 4 | 2 | 4 | 2 | 9 |
| Xanthomonadales | 11 | 40 | 0 | 0 | 4 | 0 | 0 | 1 | 0 | 0 | 0 | 0 | 0 | 0 | 0 | 0 | 0 | 0 | 0 | 0 |
| Flavobacteriales | 3 | 5 | 0 | 0 | 0 | 0 | 0 | 0 | 0 | 0 | 0 | 0 | 0 | 0 | 0 | 0 | 0 | 0 | 0 | 0 |
| Burkholderiales | 0 | 5 | 0 | 1 | 0 | 0 | 0 | 0 | 0 | 0 | 0 | 0 | 0 | 0 | 0 | 0 | 0 | 0 | 0 | 0 |
| Rhizobiales | 1 | 0 | 0 | 0 | 0 | 0 | 0 | 0 | 0 | 0 | 0 | 3 | 0 | 0 | 1 | 1 | 0 | 0 | 0 | 0 |
| Rhodospirillales | 0 | 0 | 1 | 0 | 0 | 0 | 1 | 0 | 0 | 0 | 0 | 0 | 0 | 0 | 0 | 1 | 1 | 1 | 0 | 0 |
| Caulobacterales | 1 | 2 | 0 | 0 | 0 | 0 | 0 | 0 | 0 | 0 | 0 | 0 | 0 | 0 | 0 | 0 | 0 | 0 | 0 | 0 |
| Selenomonadales | 0 | 0 | 0 | 0 | 0 | 0 | 0 | 0 | 2 | 0 | 0 | 0 | 1 | 0 | 0 | 0 | 0 | 0 | 0 | 0 |
| Pseudonocardiales | 0 | 0 | 0 | 0 | 0 | 0 | 0 | 0 | 1 | 1 | 0 | 0 | 0 | 0 | 0 | 0 | 0 | 0 | 0 | 0 |
| Acidaminococcales | 0 | 0 | 0 | 0 | 0 | 0 | 0 | 0 | 0 | 0 | 1 | 0 | 0 | 0 | 0 | 0 | 0 | 0 | 0 | 0 |
| Sphingobacteriales | 0 | 1 | 0 | 0 | 0 | 0 | 0 | 0 | 0 | 0 | 0 | 0 | 0 | 0 | 0 | 0 | 0 | 0 | 0 | 0 |
| Rhodobacterales | 0 | 0 | 0 | 0 | 0 | 0 | 0 | 0 | 0 | 0 | 0 | 0 | 0 | 0 | 0 | 1 | 0 | 0 | 0 | 0 |

C-numbered, N-numbered, and P-numbered samples are obtained from controls, Nx (5/6 nephrectomy) groups, and Nx + PAR (5/6 nephrectomy + 5% paramylon treatment) groups, respectively.
