# Supplementary material for: Renoprotective effects of paramylon, a β-1,3-D-Glucan isolated from Euglena gracilis Z in a rodent model of chronic kidney disease
Source: PLoS One. 2020 Aug 7;15(8):e0237086. doi: 10.1371/journal.pone.0237086 (PMC7413521; doi:10.1371/journal.pone.0237086)
Supplement: S5 Table — (DOCX) [file pone.0237086.s006.docx]

| Sample | C1 | C2 | N3 | N4 | N5 | N6 | P7 | P8 | P9 | P10 | C11 | C12 | N13 | N14 | N15 | N16 | P17 | P18 | P19 | P20 |
| --- | --- | --- | --- | --- | --- | --- | --- | --- | --- | --- | --- | --- | --- | --- | --- | --- | --- | --- | --- | --- |
| Rejected hit | 8801 | 10859 | 9176 | 9078 | 11351 | 5708 | 11373 | 13429 | 28200 | 21985 | 18246 | 13302 | 16188 | 15736 | 7250 | 37816 | 25784 | 16648 | 9460 | 22631 |
| Lactobacillaceae | 18461 | 10280 | 14183 | 12268 | 16172 | 11729 | 14253 | 5453 | 7278 | 5379 | 16060 | 26899 | 12830 | 17727 | 15123 | 2348 | 14484 | 15810 | 12514 | 16946 |
| Clostridiaceae | 11 | 0 | 242 | 1538 | 346 | 6232 | 16 | 5 | 0 | 271 | 6 | 9 | 374 | 4919 | 16870 | 13 | 838 | 2591 | 1822 | 7 |
| Peptostreptococcaceae | 3835 | 40 | 158 | 1028 | 353 | 2668 | 1844 | 908 | 22 | 1854 | 21 | 12 | 7044 | 1001 | 8078 | 26 | 1376 | 2088 | 2519 | 10 |
| Bifidobacteriaceae | 1381 | 50 | 2427 | 1247 | 354 | 4711 | 487 | 267 | 290 | 1403 | 22 | 296 | 197 | 3012 | 1400 | 3948 | 85 | 390 | 1440 | 86 |
| Lachnospiraceae | 147 | 94 | 189 | 113 | 167 | 42 | 505 | 1250 | 4514 | 799 | 672 | 325 | 689 | 361 | 194 | 2361 | 793 | 897 | 393 | 4578 |
| Ruminococcaceae | 107 | 1394 | 38 | 69 | 205 | 48 | 1529 | 1072 | 2989 | 1317 | 1150 | 5 | 83 | 43 | 796 | 3271 | 108 | 149 | 140 | 327 |
| Eubacteriaceae | 0 | 1 | 0 | 9 | 3 | 5 | 1242 | 1188 | 1828 | 2355 | 23 | 3 | 6 | 7 | 5 | 46 | 1088 | 0 | 1047 | 2668 |
| Akkermansiaceae | 69 | 4 | 438 | 308 | 51 | 274 | 180 | 3492 | 809 | 149 | 544 | 0 | 760 | 268 | 189 | 1036 | 307 | 84 | 625 | 704 |
| Moraxellaceae | 18 | 19 | 1 | 6152 | 312 | 90 | 194 | 0 | 0 | 0 | 0 | 0 | 0 | 0 | 2 | 11 | 0 | 0 | 0 | 1 |
| Eggerthellaceae | 175 | 223 | 115 | 72 | 112 | 76 | 252 | 224 | 436 | 287 | 279 | 106 | 263 | 167 | 429 | 1531 | 177 | 150 | 169 | 335 |
| Bacteroidaceae | 56 | 158 | 140 | 33 | 83 | 7 | 114 | 55 | 214 | 176 | 125 | 285 | 355 | 120 | 104 | 1163 | 31 | 61 | 45 | 134 |
| Streptococcaceae | 247 | 344 | 41 | 18 | 58 | 14 | 25 | 85 | 236 | 92 | 569 | 343 | 94 | 36 | 32 | 24 | 53 | 53 | 7 | 38 |
| Erysipelotrichaceae | 4 | 1 | 42 | 61 | 20 | 101 | 111 | 67 | 96 | 70 | 2 | 3 | 46 | 167 | 107 | 261 | 108 | 119 | 50 | 135 |
| f_Flintibacter_incertae_sedis | 9 | 39 | 72 | 57 | 63 | 9 | 8 | 12 | 14 | 22 | 139 | 35 | 117 | 16 | 51 | 459 | 31 | 11 | 3 | 19 |
| Micrococcaceae | 35 | 43 | 7 | 26 | 41 | 9 | 28 | 45 | 159 | 101 | 156 | 83 | 36 | 20 | 37 | 125 | 37 | 19 | 2 | 13 |
| Tannerellaceae | 42 | 17 | 39 | 23 | 31 | 5 | 40 | 10 | 20 | 32 | 34 | 23 | 38 | 10 | 47 | 470 | 8 | 4 | 13 | 7 |
| Muribaculaceae | 0 | 0 | 0 | 0 | 0 | 1 | 0 | 0 | 0 | 0 | 240 | 146 | 30 | 73 | 0 | 2 | 18 | 19 | 19 | 18 |
| Coriobacteriaceae | 3 | 2 | 9 | 23 | 8 | 4 | 39 | 53 | 37 | 41 | 26 | 16 | 22 | 10 | 12 | 40 | 34 | 45 | 43 | 69 |
| Corynebacteriaceae | 3 | 13 | 2 | 108 | 7 | 0 | 1 | 2 | 34 | 0 | 34 | 14 | 9 | 6 | 3 | 34 | 3 | 4 | 4 | 12 |
| f_Pseudoflavonifractor_incertae_sedis | 5 | 6 | 17 | 1 | 9 | 6 | 5 | 6 | 15 | 12 | 7 | 25 | 43 | 0 | 0 | 18 | 3 | 0 | 3 | 31 |
| Not determined | 0 | 0 | 0 | 0 | 0 | 0 | 99 | 103 | 2 | 0 | 0 | 0 | 0 | 1 | 0 | 1 | 0 | 0 | 0 | 4 |
| Rikenellaceae | 7 | 15 | 17 | 6 | 6 | 5 | 13 | 1 | 3 | 4 | 9 | 11 | 20 | 9 | 21 | 16 | 22 | 7 | 9 | 4 |
| f_Intestinimonas_incertae_sedis | 8 | 13 | 14 | 4 | 6 | 2 | 2 | 7 | 14 | 1 | 10 | 4 | 19 | 4 | 2 | 47 | 18 | 14 | 7 | 5 |
| Staphylococcaceae | 6 | 39 | 3 | 19 | 20 | 0 | 1 | 1 | 66 | 2 | 4 | 2 | 3 | 3 | 4 | 22 | 4 | 0 | 0 | 1 |
| Enterococcaceae | 1 | 14 | 0 | 12 | 5 | 4 | 11 | 1 | 6 | 5 | 15 | 8 | 2 | 4 | 9 | 49 | 5 | 4 | 0 | 2 |
| Enterobacteriaceae | 5 | 11 | 6 | 22 | 3 | 0 | 16 | 3 | 2 | 2 | 3 | 7 | 0 | 2 | 8 | 34 | 0 | 1 | 8 | 2 |
| Desulfovibrionaceae | 10 | 15 | 9 | 0 | 1 | 0 | 1 | 0 | 0 | 12 | 8 | 12 | 17 | 0 | 19 | 12 | 0 | 0 | 2 | 1 |
| Deferribacteraceae | 0 | 0 | 1 | 4 | 9 | 1 | 2 | 2 | 2 | 4 | 3 | 4 | 29 | 2 | 3 | 4 | 2 | 4 | 2 | 9 |
| Xanthomonadaceae | 11 | 40 | 0 | 0 | 4 | 0 | 0 | 1 | 0 | 0 | 0 | 0 | 0 | 0 | 0 | 0 | 0 | 0 | 0 | 0 |
| f_Flavonifractor_incertae_sedis | 0 | 4 | 1 | 0 | 2 | 0 | 0 | 0 | 1 | 0 | 2 | 8 | 2 | 2 | 0 | 20 | 2 | 0 | 0 | 0 |
| Aerococcaceae | 0 | 11 | 0 | 0 | 0 | 0 | 1 | 0 | 1 | 0 | 0 | 0 | 0 | 0 | 0 | 0 | 0 | 0 | 0 | 0 |
| Microbacteriaceae | 0 | 1 | 0 | 0 | 1 | 0 | 0 | 0 | 2 | 0 | 2 | 2 | 2 | 0 | 0 | 1 | 0 | 0 | 0 | 0 |
| Flavobacteriaceae | 3 | 5 | 0 | 0 | 0 | 0 | 0 | 0 | 0 | 0 | 0 | 0 | 0 | 0 | 0 | 0 | 0 | 0 | 0 | 0 |
| Catabacteriaceae | 0 | 0 | 0 | 0 | 0 | 1 | 1 | 3 | 0 | 0 | 0 | 0 | 1 | 0 | 0 | 0 | 0 | 0 | 0 | 0 |
| Bacillaceae | 0 | 0 | 0 | 0 | 0 | 0 | 0 | 1 | 3 | 1 | 0 | 0 | 0 | 0 | 0 | 0 | 0 | 0 | 0 | 1 |
| Comamonadaceae | 0 | 5 | 0 | 0 | 0 | 0 | 0 | 0 | 0 | 0 | 0 | 0 | 0 | 0 | 0 | 0 | 0 | 0 | 0 | 0 |
| f_Reyranella_incertae_sedis | 0 | 0 | 1 | 0 | 0 | 0 | 1 | 0 | 0 | 0 | 0 | 0 | 0 | 0 | 0 | 1 | 1 | 1 | 0 | 0 |
| Caulobacteraceae | 1 | 2 | 0 | 0 | 0 | 0 | 0 | 0 | 0 | 0 | 0 | 0 | 0 | 0 | 0 | 0 | 0 | 0 | 0 | 0 |
| Selenomonadaceae | 0 | 0 | 0 | 0 | 0 | 0 | 0 | 0 | 2 | 0 | 0 | 0 | 1 | 0 | 0 | 0 | 0 | 0 | 0 | 0 |
| Rhizobiaceae | 0 | 0 | 0 | 0 | 0 | 0 | 0 | 0 | 0 | 0 | 0 | 3 | 0 | 0 | 0 | 0 | 0 | 0 | 0 | 0 |
| Morganellaceae | 0 | 0 | 0 | 0 | 0 | 0 | 0 | 0 | 0 | 1 | 1 | 0 | 0 | 0 | 0 | 0 | 0 | 0 | 0 | 1 |
| Pseudonocardiaceae | 0 | 0 | 0 | 0 | 0 | 0 | 0 | 0 | 1 | 1 | 0 | 0 | 0 | 0 | 0 | 0 | 0 | 0 | 0 | 0 |
| Bradyrhizobiaceae | 0 | 0 | 0 | 0 | 0 | 0 | 0 | 0 | 0 | 0 | 0 | 0 | 0 | 0 | 1 | 1 | 0 | 0 | 0 | 0 |
| Thermoactinomycetaceae | 0 | 0 | 0 | 0 | 0 | 0 | 0 | 0 | 1 | 1 | 0 | 0 | 0 | 0 | 0 | 0 | 0 | 0 | 0 | 0 |
| Rhodobacteraceae | 0 | 0 | 0 | 0 | 0 | 0 | 0 | 0 | 0 | 0 | 0 | 0 | 0 | 0 | 0 | 1 | 0 | 0 | 0 | 0 |
| Methylobacteriaceae | 1 | 0 | 0 | 0 | 0 | 0 | 0 | 0 | 0 | 0 | 0 | 0 | 0 | 0 | 0 | 0 | 0 | 0 | 0 | 0 |
| Sphingobacteriaceae | 0 | 1 | 0 | 0 | 0 | 0 | 0 | 0 | 0 | 0 | 0 | 0 | 0 | 0 | 0 | 0 | 0 | 0 | 0 | 0 |
| Pseudomonadaceae | 0 | 0 | 0 | 1 | 0 | 0 | 0 | 0 | 0 | 0 | 0 | 0 | 0 | 0 | 0 | 0 | 0 | 0 | 0 | 0 |
| Prevotellaceae | 0 | 0 | 0 | 0 | 0 | 0 | 0 | 0 | 0 | 0 | 0 | 0 | 0 | 0 | 1 | 0 | 0 | 0 | 0 | 0 |
| Christensenellaceae | 0 | 0 | 0 | 0 | 0 | 0 | 1 | 0 | 0 | 0 | 0 | 0 | 0 | 0 | 0 | 0 | 0 | 0 | 0 | 0 |
| Dermacoccaceae | 0 | 0 | 0 | 0 | 0 | 0 | 0 | 0 | 0 | 0 | 0 | 0 | 0 | 0 | 0 | 0 | 0 | 1 | 0 | 0 |
| Acidaminococcaceae | 0 | 0 | 0 | 0 | 0 | 0 | 0 | 0 | 0 | 0 | 1 | 0 | 0 | 0 | 0 | 0 | 0 | 0 | 0 | 0 |
| Oxalobacteraceae | 0 | 0 | 0 | 1 | 0 | 0 | 0 | 0 | 0 | 0 | 0 | 0 | 0 | 0 | 0 | 0 | 0 | 0 | 0 | 0 |
| Odoribacteraceae | 0 | 0 | 0 | 0 | 1 | 0 | 0 | 0 | 0 | 0 | 0 | 0 | 0 | 0 | 0 | 0 | 0 | 0 | 0 | 0 |
| Sanguibacteraceae | 0 | 0 | 0 | 0 | 0 | 0 | 0 | 0 | 1 | 0 | 0 | 0 | 0 | 0 | 0 | 0 | 0 | 0 | 0 | 0 |
| Paenibacillaceae | 0 | 0 | 0 | 0 | 1 | 0 | 0 | 0 | 0 | 0 | 0 | 0 | 0 | 0 | 0 | 0 | 0 | 0 | 0 | 0 |

C-numbered, N-numbered, and P-numbered samples are obtained from controls, Nx (5/6 nephrectomy) groups, and Nx + PAR (5/6 nephrectomy + 5% paramylon treatment) groups, respectively.
