# Supplementary material for: Renoprotective effects of paramylon, a β-1,3-D-Glucan isolated from Euglena gracilis Z in a rodent model of chronic kidney disease
Source: PLoS One. 2020 Aug 7;15(8):e0237086. doi: 10.1371/journal.pone.0237086 (PMC7413521; doi:10.1371/journal.pone.0237086)
Supplement: S6 Table — (DOCX) [file pone.0237086.s007.docx]

| Sample | C1 | C2 | C11 | C12 | N3 | N4 | N5 | N6 | N13 | N14 | N15 | N16 | P7 | P8 | P9 | P10 | P17 | P18 | P19 | P20 |
| --- | --- | --- | --- | --- | --- | --- | --- | --- | --- | --- | --- | --- | --- | --- | --- | --- | --- | --- | --- | --- |
| Rejected hit | 8801 | 10859 | 18246 | 13302 | 9176 | 9078 | 11351 | 5708 | 16188 | 15736 | 7250 | 37816 | 11373 | 13429 | 28200 | 21985 | 25784 | 16648 | 9460 | 22631 |
| Lactobacillus | 18461 | 10280 | 16060 | 26899 | 14183 | 12268 | 16172 | 11729 | 12830 | 17727 | 15123 | 2348 | 14253 | 5453 | 7278 | 5379 | 14484 | 15810 | 12514 | 16945 |
| % | 55.17004 | 43.26053 | 41.80876 | 64.05897 | 51.78545 | 37.98025 | 54.25935 | 36.93941 | 32.6297 | 40.5411 | 29.77144 | 4.252699 | 43.99753 | 19.65328 | 15.38754 | 14.786 | 31.88904 | 40.3584 | 41.23772 | 34.72407 |
| Clostridium | 11 | 0 | 6 | 9 | 242 | 1538 | 346 | 6232 | 374 | 4919 | 16870 | 12 | 14 | 2 | 0 | 271 | 837 | 2591 | 1822 | 6 |
| % | 0.032873 | 0 | 0.01562 | 0.021433 | 0.883599 | 4.761462 | 1.160879 | 19.62711 | 0.95117 | 11.2496 | 33.21062 | 0.021734 | 0.043217 | 0.007208 | 0 | 0.744935 | 1.842801 | 6.614081 | 6.004086 | 0.012295 |
| Romboutsia | 3835 | 39 | 21 | 12 | 158 | 1028 | 353 | 2668 | 7044 | 1001 | 8078 | 26 | 1844 | 907 | 21 | 1854 | 1375 | 2088 | 2519 | 8 |
| % | 11.46076 | 0.164121 | 0.054669 | 0.028578 | 0.576895 | 3.182564 | 1.184365 | 8.40262 | 17.91455 | 2.289256 | 15.90251 | 0.047091 | 5.692236 | 3.26894 | 0.044399 | 5.096347 | 3.027301 | 5.330066 | 8.300929 | 0.016394 |
| Bifidobacterium | 1381 | 50 | 22 | 296 | 2427 | 1247 | 354 | 4711 | 197 | 3012 | 1400 | 3948 | 487 | 267 | 290 | 1403 | 85 | 390 | 1440 | 86 |
| % | 4.12707 | 0.210411 | 0.057272 | 0.704913 | 8.861545 | 3.860562 | 1.18772 | 14.83686 | 0.501017 | 6.88835 | 2.756068 | 7.150619 | 1.503318 | 0.962301 | 0.613134 | 3.856621 | 0.187142 | 0.995558 | 4.745271 | 0.176233 |
| Eubacterium | 0 | 1 | 23 | 3 | 0 | 9 | 3 | 5 | 6 | 7 | 5 | 46 | 1242 | 1188 | 1828 | 2355 | 1088 | 0 | 1047 | 2668 |
| % | 0 | 0.004208 | 0.059876 | 0.007144 | 0 | 0.027863 | 0.010065 | 0.015747 | 0.015259 | 0.016009 | 0.009843 | 0.083315 | 3.833925 | 4.281698 | 3.864857 | 6.473515 | 2.395421 | 0 | 3.450208 | 5.467325 |
| Ruminococcus | 93 | 1388 | 963 | 0 | 34 | 59 | 157 | 44 | 49 | 7 | 739 | 2719 | 853 | 613 | 1241 | 1294 | 35 | 79 | 11 | 171 |
| % | 0.277927 | 5.841013 | 2.506964 | 0 | 0.124142 | 0.182657 | 0.526757 | 0.138574 | 0.124619 | 0.016009 | 1.45481 | 4.924654 | 2.633122 | 2.209327 | 2.62379 | 3.556997 | 0.077059 | 0.201664 | 0.036249 | 0.350417 |
| Akkermansia | 69 | 4 | 544 | 0 | 438 | 308 | 51 | 274 | 760 | 268 | 189 | 1036 | 180 | 3492 | 809 | 149 | 307 | 84 | 625 | 704 |
| % | 0.206204 | 0.016833 | 1.416187 | 0 | 1.599241 | 0.953531 | 0.171112 | 0.862938 | 1.932859 | 0.612908 | 0.372069 | 1.876404 | 0.555641 | 12.5856 | 1.710432 | 0.409577 | 0.675914 | 0.214428 | 2.05958 | 1.442653 |
| Murimonas | 5 | 5 | 1 | 2 | 6 | 3 | 1 | 0 | 13 | 10 | 8 | 489 | 80 | 589 | 3554 | 102 | 270 | 318 | 134 | 2075 |
| % | 0.014942 | 0.021041 | 0.002603 | 0.004763 | 0.021907 | 0.009288 | 0.003355 | 0 | 0.033062 | 0.02287 | 0.015749 | 0.885677 | 0.246952 | 2.122829 | 7.51406 | 0.280382 | 0.594452 | 0.811763 | 0.441574 | 4.252136 |
| Acinetobacter | 18 | 19 | 0 | 0 | 1 | 6152 | 312 | 90 | 0 | 0 | 2 | 11 | 194 | 0 | 0 | 0 | 0 | 0 | 0 | 1 |
| % | 0.053792 | 0.079956 | 0 | 0 | 0.003651 | 19.04585 | 1.046804 | 0.283447 | 0 | 0 | 0.003937 | 0.019923 | 0.598858 | 0 | 0 | 0 | 0 | 0 | 0 | 0.002049 |
| Blautia | 8 | 28 | 135 | 92 | 31 | 26 | 13 | 19 | 22 | 174 | 28 | 1274 | 260 | 262 | 343 | 107 | 216 | 236 | 137 | 1801 |
| % | 0.023908 | 0.11783 | 0.351444 | 0.219095 | 0.113188 | 0.080493 | 0.043617 | 0.059839 | 0.055951 | 0.397933 | 0.055121 | 2.307469 | 0.802593 | 0.94428 | 0.725189 | 0.294126 | 0.475561 | 0.60244 | 0.45146 | 3.690649 |
| Enterorhabdus | 172 | 223 | 276 | 99 | 90 | 37 | 83 | 47 | 223 | 152 | 268 | 1142 | 214 | 191 | 291 | 203 | 106 | 114 | 97 | 154 |
| % | 0.514016 | 0.938434 | 0.718507 | 0.235765 | 0.328611 | 0.114548 | 0.278477 | 0.148022 | 0.567141 | 0.347619 | 0.52759 | 2.068391 | 0.660596 | 0.688388 | 0.615248 | 0.558014 | 0.233377 | 0.291009 | 0.319647 | 0.31558 |
| Acutalibacter | 11 | 4 | 184 | 5 | 4 | 9 | 42 | 4 | 21 | 31 | 51 | 529 | 666 | 454 | 1738 | 18 | 48 | 67 | 122 | 142 |
| % | 0.032873 | 0.016833 | 0.479005 | 0.011907 | 0.014605 | 0.027863 | 0.140916 | 0.012598 | 0.053408 | 0.070896 | 0.1004 | 0.958125 | 2.055873 | 1.636272 | 3.674574 | 0.049479 | 0.10568 | 0.171032 | 0.40203 | 0.29099 |
| Bacteroides | 56 | 158 | 125 | 285 | 140 | 33 | 83 | 7 | 355 | 120 | 104 | 1163 | 114 | 55 | 214 | 176 | 31 | 61 | 45 | 134 |
| % | 0.167354 | 0.664899 | 0.325411 | 0.678717 | 0.511173 | 0.102164 | 0.278477 | 0.022046 | 0.902848 | 0.274436 | 0.204737 | 2.106426 | 0.351906 | 0.198227 | 0.45245 | 0.483796 | 0.068252 | 0.155716 | 0.14829 | 0.274596 |
| g_Lachnospiraceae bacterium KNHs209_incertae_sedis | 45 | 12 | 219 | 68 | 90 | 7 | 86 | 3 | 580 | 82 | 58 | 56 | 39 | 7 | 63 | 103 | 39 | 96 | 25 | 127 |
| % | 0.134481 | 0.050499 | 0.570119 | 0.161939 | 0.328611 | 0.021671 | 0.288542 | 0.009448 | 1.475076 | 0.187531 | 0.11418 | 0.101427 | 0.120389 | 0.025229 | 0.133198 | 0.28313 | 0.085865 | 0.24506 | 0.082383 | 0.260251 |
| Lactococcus | 213 | 287 | 477 | 236 | 37 | 15 | 52 | 14 | 69 | 23 | 32 | 1 | 17 | 78 | 88 | 19 | 28 | 26 | 3 | 7 |
| % | 0.636543 | 1.20776 | 1.241767 | 0.562025 | 0.135096 | 0.046438 | 0.174467 | 0.044092 | 0.175483 | 0.0526 | 0.062996 | 0.001811 | 0.052477 | 0.281122 | 0.186054 | 0.052228 | 0.061647 | 0.066371 | 0.009886 | 0.014345 |
| Lachnoclostridium | 22 | 23 | 104 | 29 | 14 | 33 | 22 | 5 | 39 | 8 | 6 | 100 | 39 | 207 | 259 | 297 | 33 | 26 | 16 | 44 |
| % | 0.065746 | 0.096789 | 0.270742 | 0.069062 | 0.051117 | 0.102164 | 0.073813 | 0.015747 | 0.099186 | 0.018296 | 0.011812 | 0.18112 | 0.120389 | 0.746053 | 0.547592 | 0.816405 | 0.072655 | 0.066371 | 0.052725 | 0.090166 |
| Flintibacter | 9 | 39 | 139 | 35 | 72 | 57 | 63 | 9 | 117 | 16 | 51 | 459 | 8 | 12 | 14 | 22 | 31 | 11 | 3 | 19 |
| % | 0.026896 | 0.164121 | 0.361857 | 0.083351 | 0.262889 | 0.176465 | 0.211374 | 0.028345 | 0.297558 | 0.036592 | 0.1004 | 0.831341 | 0.024695 | 0.043249 | 0.0296 | 0.060474 | 0.068252 | 0.02808 | 0.009886 | 0.038935 |
| Not determined | 4 | 4 | 5 | 3 | 5 | 10 | 10 | 5 | 12 | 9 | 101 | 286 | 110 | 122 | 55 | 26 | 83 | 32 | 75 | 223 |
| Faecalibaculum | 4 | 1 | 2 | 3 | 41 | 61 | 20 | 100 | 39 | 138 | 107 | 95 | 104 | 67 | 50 | 50 | 94 | 93 | 29 | 78 |
| % | 0.011954 | 0.004208 | 0.005207 | 0.007144 | 0.149701 | 0.188849 | 0.067103 | 0.314941 | 0.099186 | 0.315602 | 0.210642 | 0.172064 | 0.321037 | 0.241476 | 0.105713 | 0.137442 | 0.206957 | 0.237402 | 0.095564 | 0.159839 |
| Anaerotaenia | 58 | 21 | 177 | 125 | 27 | 36 | 31 | 8 | 17 | 59 | 84 | 172 | 32 | 9 | 45 | 53 | 21 | 23 | 18 | 65 |
| % | 0.173331 | 0.088373 | 0.460782 | 0.297683 | 0.098583 | 0.111452 | 0.104009 | 0.025195 | 0.043235 | 0.134931 | 0.165364 | 0.311526 | 0.098781 | 0.032437 | 0.095141 | 0.145688 | 0.046235 | 0.058712 | 0.059316 | 0.133199 |
| Parabacteroides | 42 | 17 | 34 | 23 | 39 | 23 | 31 | 5 | 38 | 10 | 47 | 470 | 40 | 10 | 20 | 32 | 8 | 4 | 13 | 7 |
| % | 0.125516 | 0.07154 | 0.088512 | 0.054774 | 0.142398 | 0.071205 | 0.104009 | 0.015747 | 0.096643 | 0.02287 | 0.092525 | 0.851264 | 0.123476 | 0.036041 | 0.042285 | 0.087963 | 0.017613 | 0.010211 | 0.042839 | 0.014345 |
| Rothia | 34 | 43 | 153 | 80 | 7 | 9 | 35 | 7 | 34 | 20 | 0 | 71 | 28 | 45 | 159 | 101 | 37 | 19 | 2 | 13 |
| % | 0.101608 | 0.180954 | 0.398303 | 0.190517 | 0.025559 | 0.027863 | 0.11743 | 0.022046 | 0.08647 | 0.045739 | 0 | 0.128595 | 0.086433 | 0.162186 | 0.336166 | 0.277633 | 0.081462 | 0.048502 | 0.006591 | 0.02664 |
| Streptococcus | 34 | 57 | 92 | 107 | 4 | 3 | 6 | 0 | 25 | 13 | 0 | 23 | 8 | 7 | 148 | 73 | 25 | 27 | 4 | 31 |
| % | 0.101608 | 0.239869 | 0.239502 | 0.254817 | 0.014605 | 0.009288 | 0.020131 | 0 | 0.063581 | 0.029731 | 0 | 0.041658 | 0.024695 | 0.025229 | 0.31291 | 0.200665 | 0.055042 | 0.068923 | 0.013181 | 0.063526 |
| Asaccharobacter | 3 | 0 | 3 | 7 | 21 | 31 | 24 | 25 | 30 | 13 | 62 | 118 | 35 | 28 | 129 | 69 | 3 | 12 | 5 | 6 |
| % | 0.008965 | 0 | 0.00781 | 0.01667 | 0.076676 | 0.095972 | 0.080523 | 0.078735 | 0.076297 | 0.029731 | 0.122054 | 0.213722 | 0.108041 | 0.100915 | 0.272739 | 0.18967 | 0.006605 | 0.030633 | 0.016477 | 0.012295 |
| Frisingicoccus | 1 | 0 | 0 | 0 | 0 | 1 | 0 | 0 | 0 | 0 | 0 | 133 | 11 | 10 | 29 | 20 | 48 | 32 | 26 | 281 |
| % | 0.002988 | 0 | 0 | 0 | 0 | 0.003096 | 0 | 0 | 0 | 0 | 0 | 0.24089 | 0.033956 | 0.036041 | 0.061313 | 0.054977 | 0.10568 | 0.081687 | 0.085679 | 0.575831 |
| Muribaculum | 0 | 0 | 240 | 146 | 0 | 0 | 0 | 1 | 30 | 73 | 0 | 2 | 0 | 0 | 0 | 0 | 18 | 19 | 19 | 18 |
| % | 0 | 0 | 0.624788 | 0.347694 | 0 | 0 | 0 | 0.003149 | 0.076297 | 0.166949 | 0 | 0.003622 | 0 | 0 | 0 | 0 | 0.03963 | 0.048502 | 0.062611 | 0.036886 |
| Marvinbryantia | 0 | 0 | 0 | 0 | 1 | 0 | 0 | 0 | 0 | 1 | 0 | 23 | 12 | 134 | 169 | 80 | 40 | 49 | 7 | 46 |
| % | 0 | 0 | 0 | 0 | 0.003651 | 0 | 0 | 0 | 0 | 0.002287 | 0 | 0.041658 | 0.037043 | 0.482952 | 0.357309 | 0.219907 | 0.088067 | 0.125083 | 0.023067 | 0.094264 |
| Parvibacter | 3 | 2 | 26 | 16 | 9 | 22 | 8 | 4 | 22 | 10 | 12 | 40 | 39 | 44 | 37 | 41 | 34 | 45 | 34 | 46 |
| % | 0.008965 | 0.008416 | 0.067685 | 0.038103 | 0.032861 | 0.068109 | 0.026841 | 0.012598 | 0.055951 | 0.02287 | 0.023623 | 0.072448 | 0.120389 | 0.158581 | 0.078227 | 0.112702 | 0.074857 | 0.114872 | 0.112041 | 0.094264 |
| Coprococcus | 0 | 0 | 1 | 0 | 0 | 0 | 0 | 0 | 2 | 6 | 2 | 13 | 7 | 1 | 3 | 14 | 91 | 90 | 16 | 67 |
| % | 0 | 0 | 0.002603 | 0 | 0 | 0 | 0 | 0 | 0.005086 | 0.013722 | 0.003937 | 0.023546 | 0.021608 | 0.003604 | 0.006343 | 0.038484 | 0.200352 | 0.229744 | 0.052725 | 0.137298 |
| Corynebacterium | 3 | 13 | 34 | 14 | 2 | 108 | 7 | 0 | 9 | 6 | 3 | 34 | 1 | 2 | 34 | 0 | 3 | 4 | 4 | 12 |
| % | 0.008965 | 0.054707 | 0.088512 | 0.03334 | 0.007302 | 0.334355 | 0.023486 | 0 | 0.022889 | 0.013722 | 0.005906 | 0.061581 | 0.003087 | 0.007208 | 0.071885 | 0 | 0.006605 | 0.010211 | 0.013181 | 0.024591 |
| Erysipelatoclostridium | 0 | 0 | 0 | 0 | 0 | 0 | 0 | 0 | 1 | 23 | 0 | 160 | 1 | 0 | 3 | 20 | 1 | 11 | 17 | 42 |
| % | 0 | 0 | 0 | 0 | 0 | 0 | 0 | 0 | 0.002543 | 0.0526 | 0 | 0.289792 | 0.003087 | 0 | 0.006343 | 0.054977 | 0.002202 | 0.02808 | 0.056021 | 0.086067 |
| Pseudoflavonifractor | 5 | 6 | 7 | 25 | 17 | 1 | 9 | 6 | 43 | 0 | 0 | 18 | 5 | 6 | 15 | 12 | 3 | 0 | 3 | 31 |
| % | 0.014942 | 0.025249 | 0.018223 | 0.059537 | 0.062071 | 0.003096 | 0.030196 | 0.018896 | 0.109359 | 0 | 0 | 0.032602 | 0.015434 | 0.021625 | 0.031714 | 0.032986 | 0.006605 | 0 | 0.009886 | 0.063526 |
| Alistipes | 7 | 15 | 9 | 11 | 17 | 6 | 6 | 5 | 20 | 9 | 21 | 16 | 13 | 1 | 3 | 4 | 22 | 7 | 9 | 4 |
| % | 0.020919 | 0.063123 | 0.02343 | 0.026196 | 0.062071 | 0.018575 | 0.020131 | 0.015747 | 0.050865 | 0.020583 | 0.041341 | 0.028979 | 0.04013 | 0.003604 | 0.006343 | 0.010995 | 0.048437 | 0.017869 | 0.029658 | 0.008197 |
| Intestinimonas | 8 | 13 | 10 | 4 | 14 | 4 | 6 | 2 | 19 | 4 | 2 | 47 | 2 | 7 | 14 | 1 | 18 | 14 | 7 | 5 |
| % | 0.023908 | 0.054707 | 0.026033 | 0.009526 | 0.051117 | 0.012384 | 0.020131 | 0.006299 | 0.048321 | 0.009148 | 0.003937 | 0.085126 | 0.006174 | 0.025229 | 0.0296 | 0.002749 | 0.03963 | 0.035738 | 0.023067 | 0.010246 |
| Enterococcus | 1 | 14 | 15 | 8 | 0 | 12 | 5 | 4 | 1 | 4 | 8 | 45 | 11 | 1 | 6 | 5 | 5 | 4 | 0 | 2 |
| % | 0.002988 | 0.058915 | 0.039049 | 0.019052 | 0 | 0.037151 | 0.016776 | 0.012598 | 0.002543 | 0.009148 | 0.015749 | 0.081504 | 0.033956 | 0.003604 | 0.012686 | 0.013744 | 0.011008 | 0.010211 | 0 | 0.004098 |
| Escherichia | 5 | 11 | 3 | 7 | 6 | 11 | 3 | 0 | 0 | 2 | 8 | 34 | 16 | 3 | 2 | 2 | 0 | 1 | 8 | 2 |
| % | 0.014942 | 0.04629 | 0.00781 | 0.01667 | 0.021907 | 0.034055 | 0.010065 | 0 | 0 | 0.004574 | 0.015749 | 0.061581 | 0.04939 | 0.010812 | 0.004229 | 0.005498 | 0 | 0.002553 | 0.026363 | 0.004098 |
| Desulfovibrio | 10 | 15 | 8 | 12 | 9 | 0 | 1 | 0 | 17 | 0 | 19 | 12 | 1 | 0 | 0 | 12 | 0 | 0 | 2 | 1 |
| % | 0.029885 | 0.063123 | 0.020826 | 0.028578 | 0.032861 | 0 | 0.003355 | 0 | 0.043235 | 0 | 0.037404 | 0.021734 | 0.003087 | 0 | 0 | 0.032986 | 0 | 0 | 0.006591 | 0.002049 |
| Roseburia | 3 | 0 | 24 | 0 | 1 | 1 | 8 | 5 | 3 | 1 | 5 | 8 | 14 | 15 | 8 | 1 | 3 | 7 | 2 | 0 |
| % | 0.008965 | 0 | 0.062479 | 0 | 0.003651 | 0.003096 | 0.026841 | 0.015747 | 0.00763 | 0.002287 | 0.009843 | 0.01449 | 0.043217 | 0.054062 | 0.016914 | 0.002749 | 0.006605 | 0.017869 | 0.006591 | 0 |
| Staphylococcus | 6 | 23 | 3 | 2 | 3 | 6 | 20 | 0 | 2 | 0 | 1 | 14 | 0 | 1 | 20 | 2 | 0 | 0 | 0 | 1 |
| % | 0.017931 | 0.096789 | 0.00781 | 0.004763 | 0.010954 | 0.018575 | 0.067103 | 0 | 0.005086 | 0 | 0.001969 | 0.025357 | 0 | 0.003604 | 0.042285 | 0.005498 | 0 | 0 | 0 | 0.002049 |
| Jeotgalicoccus | 0 | 16 | 1 | 0 | 0 | 13 | 0 | 0 | 1 | 3 | 3 | 8 | 1 | 0 | 46 | 0 | 4 | 0 | 0 | 0 |
| % | 0 | 0.067332 | 0.002603 | 0 | 0 | 0.040246 | 0 | 0 | 0.002543 | 0.006861 | 0.005906 | 0.01449 | 0.003087 | 0 | 0.097256 | 0 | 0.008807 | 0 | 0 | 0 |
| Mucispirillum | 0 | 0 | 3 | 4 | 1 | 4 | 9 | 1 | 29 | 2 | 3 | 4 | 2 | 2 | 2 | 4 | 2 | 4 | 2 | 9 |
| % | 0 | 0 | 0.00781 | 0.009526 | 0.003651 | 0.012384 | 0.030196 | 0.003149 | 0.073754 | 0.004574 | 0.005906 | 0.007245 | 0.006174 | 0.007208 | 0.004229 | 0.010995 | 0.004403 | 0.010211 | 0.006591 | 0.018443 |
| Tyzzerella | 0 | 0 | 1 | 1 | 16 | 1 | 0 | 1 | 2 | 0 | 0 | 50 | 0 | 0 | 0 | 0 | 5 | 1 | 0 | 0 |
| % | 0 | 0 | 0.002603 | 0.002381 | 0.05842 | 0.003096 | 0 | 0.003149 | 0.005086 | 0 | 0 | 0.09056 | 0 | 0 | 0 | 0 | 0.011008 | 0.002553 | 0 | 0 |
| Kocuria | 0 | 0 | 2 | 2 | 0 | 0 | 0 | 0 | 1 | 0 | 35 | 27 | 0 | 0 | 0 | 0 | 0 | 0 | 0 | 0 |
| % | 0 | 0 | 0.005207 | 0.004763 | 0 | 0 | 0 | 0 | 0.002543 | 0 | 0.068902 | 0.048902 | 0 | 0 | 0 | 0 | 0 | 0 | 0 | 0 |
| Ruthenibacterium | 1 | 0 | 0 | 0 | 0 | 0 | 2 | 0 | 10 | 3 | 5 | 0 | 9 | 0 | 0 | 2 | 21 | 3 | 6 | 1 |
| % | 0.002988 | 0 | 0 | 0 | 0 | 0 | 0.00671 | 0 | 0.025432 | 0.006861 | 0.009843 | 0 | 0.027782 | 0 | 0 | 0.005498 | 0.046235 | 0.007658 | 0.019772 | 0.002049 |
| Harryflintia | 2 | 2 | 3 | 0 | 0 | 0 | 4 | 0 | 3 | 1 | 0 | 20 | 0 | 4 | 6 | 3 | 4 | 0 | 1 | 8 |
| % | 0.005977 | 0.008416 | 0.00781 | 0 | 0 | 0 | 0.013421 | 0 | 0.00763 | 0.002287 | 0 | 0.036224 | 0 | 0.014416 | 0.012686 | 0.008247 | 0.008807 | 0 | 0.003295 | 0.016394 |
| Stenotrophomonas | 11 | 40 | 0 | 0 | 0 | 0 | 4 | 0 | 0 | 0 | 0 | 0 | 0 | 1 | 0 | 0 | 0 | 0 | 0 | 0 |
| % | 0.032873 | 0.168329 | 0 | 0 | 0 | 0 | 0.013421 | 0 | 0 | 0 | 0 | 0 | 0 | 0.003604 | 0 | 0 | 0 | 0 | 0 | 0 |
| Longicatena | 0 | 0 | 0 | 0 | 0 | 0 | 0 | 0 | 0 | 0 | 0 | 0 | 0 | 0 | 43 | 0 | 1 | 0 | 0 | 3 |
| % | 0 | 0 | 0 | 0 | 0 | 0 | 0 | 0 | 0 | 0 | 0 | 0 | 0 | 0 | 0.090913 | 0 | 0.002202 | 0 | 0 | 0.006148 |
| Flavonifractor | 0 | 4 | 2 | 8 | 1 | 0 | 2 | 0 | 2 | 2 | 0 | 20 | 0 | 0 | 1 | 0 | 2 | 0 | 0 | 0 |
| % | 0 | 0.016833 | 0.005207 | 0.019052 | 0.003651 | 0 | 0.00671 | 0 | 0.005086 | 0.004574 | 0 | 0.036224 | 0 | 0 | 0.002114 | 0 | 0.004403 | 0 | 0 | 0 |
| Arthrobacter | 1 | 0 | 1 | 1 | 0 | 0 | 6 | 2 | 1 | 0 | 2 | 27 | 0 | 0 | 0 | 0 | 0 | 0 | 0 | 0 |
| % | 0.002988 | 0 | 0.002603 | 0.002381 | 0 | 0 | 0.020131 | 0.006299 | 0.002543 | 0 | 0.003937 | 0.048902 | 0 | 0 | 0 | 0 | 0 | 0 | 0 | 0 |
| Enorma | 0 | 0 | 0 | 0 | 0 | 0 | 0 | 0 | 0 | 0 | 0 | 0 | 0 | 9 | 0 | 0 | 0 | 0 | 9 | 23 |
| % | 0 | 0 | 0 | 0 | 0 | 0 | 0 | 0 | 0 | 0 | 0 | 0 | 0 | 0.032437 | 0 | 0 | 0 | 0 | 0.029658 | 0.047132 |
| Holdemania | 0 | 0 | 0 | 0 | 0 | 0 | 0 | 0 | 1 | 0 | 0 | 6 | 6 | 0 | 0 | 0 | 3 | 4 | 4 | 11 |
| % | 0 | 0 | 0 | 0 | 0 | 0 | 0 | 0 | 0.002543 | 0 | 0 | 0.010867 | 0.018521 | 0 | 0 | 0 | 0.006605 | 0.010211 | 0.013181 | 0.022541 |
| Turicibacter | 0 | 0 | 0 | 0 | 1 | 0 | 0 | 1 | 5 | 6 | 0 | 0 | 0 | 0 | 0 | 0 | 9 | 11 | 0 | 1 |
| % | 0 | 0 | 0 | 0 | 0.003651 | 0 | 0 | 0.003149 | 0.012716 | 0.013722 | 0 | 0 | 0 | 0 | 0 | 0 | 0.019815 | 0.02808 | 0 | 0.002049 |
| Dorea | 0 | 0 | 1 | 0 | 0 | 2 | 0 | 0 | 0 | 0 | 0 | 3 | 1 | 0 | 0 | 1 | 0 | 7 | 0 | 8 |
| % | 0 | 0 | 0.002603 | 0 | 0 | 0.006192 | 0 | 0 | 0 | 0 | 0 | 0.005434 | 0.003087 | 0 | 0 | 0.002749 | 0 | 0.017869 | 0 | 0.016394 |
| Robinsoniella | 0 | 0 | 0 | 0 | 0 | 0 | 0 | 0 | 0 | 0 | 0 | 0 | 0 | 1 | 2 | 1 | 3 | 1 | 2 | 11 |
| % | 0 | 0 | 0 | 0 | 0 | 0 | 0 | 0 | 0 | 0 | 0 | 0 | 0 | 0.003604 | 0.004229 | 0.002749 | 0.006605 | 0.002553 | 0.006591 | 0.022541 |
| Acetatifactor | 0 | 0 | 0 | 1 | 1 | 0 | 1 | 0 | 3 | 0 | 0 | 8 | 0 | 0 | 0 | 0 | 3 | 1 | 0 | 0 |
| % | 0 | 0 | 0 | 0.002381 | 0.003651 | 0 | 0.003355 | 0 | 0.00763 | 0 | 0 | 0.01449 | 0 | 0 | 0 | 0 | 0.006605 | 0.002553 | 0 | 0 |
| Glutamicibacter | 0 | 0 | 0 | 0 | 0 | 17 | 0 | 0 | 0 | 0 | 0 | 0 | 0 | 0 | 0 | 0 | 0 | 0 | 0 | 0 |
| % | 0 | 0 | 0 | 0 | 0 | 0.05263 | 0 | 0 | 0 | 0 | 0 | 0 | 0 | 0 | 0 | 0 | 0 | 0 | 0 | 0 |
| Adlercreutzia | 0 | 0 | 0 | 0 | 1 | 1 | 0 | 0 | 1 | 0 | 1 | 4 | 0 | 0 | 2 | 3 | 0 | 0 | 1 | 2 |
| % | 0 | 0 | 0 | 0 | 0.003651 | 0.003096 | 0 | 0 | 0.002543 | 0 | 0.001969 | 0.007245 | 0 | 0 | 0.004229 | 0.008247 | 0 | 0 | 0.003295 | 0.004098 |
| Anaerobium | 0 | 0 | 1 | 0 | 0 | 0 | 0 | 0 | 0 | 0 | 0 | 7 | 0 | 0 | 0 | 2 | 1 | 0 | 1 | 4 |
| % | 0 | 0 | 0.002603 | 0 | 0 | 0 | 0 | 0 | 0 | 0 | 0 | 0.012678 | 0 | 0 | 0 | 0.005498 | 0.002202 | 0 | 0.003295 | 0.008197 |
| Anaerostipes | 0 | 0 | 0 | 0 | 0 | 2 | 0 | 0 | 0 | 11 | 0 | 1 | 0 | 1 | 0 | 0 | 0 | 0 | 0 | 0 |
| % | 0 | 0 | 0 | 0 | 0 | 0.006192 | 0 | 0 | 0 | 0.025157 | 0 | 0.001811 | 0 | 0.003604 | 0 | 0 | 0 | 0 | 0 | 0 |
| Muricomes | 0 | 0 | 1 | 1 | 0 | 0 | 0 | 0 | 3 | 3 | 0 | 2 | 1 | 0 | 0 | 1 | 1 | 1 | 0 | 1 |
| % | 0 | 0 | 0.002603 | 0.002381 | 0 | 0 | 0 | 0 | 0.00763 | 0.006861 | 0 | 0.003622 | 0.003087 | 0 | 0 | 0.002749 | 0.002202 | 0.002553 | 0 | 0.002049 |
| Aerococcus | 0 | 11 | 0 | 0 | 0 | 0 | 0 | 0 | 0 | 0 | 0 | 0 | 1 | 0 | 0 | 0 | 0 | 0 | 0 | 0 |
| % | 0 | 0.04629 | 0 | 0 | 0 | 0 | 0 | 0 | 0 | 0 | 0 | 0 | 0.003087 | 0 | 0 | 0 | 0 | 0 | 0 | 0 |
| Faecalicatena | 1 | 0 | 0 | 0 | 0 | 0 | 0 | 0 | 1 | 0 | 0 | 3 | 1 | 0 | 1 | 2 | 0 | 1 | 0 | 1 |
| % | 0.002988 | 0 | 0 | 0 | 0 | 0 | 0 | 0 | 0.002543 | 0 | 0 | 0.005434 | 0.003087 | 0 | 0.002114 | 0.005498 | 0 | 0.002553 | 0 | 0.002049 |
| Ruminiclostridium | 0 | 0 | 0 | 0 | 0 | 0 | 0 | 0 | 0 | 1 | 0 | 0 | 1 | 1 | 3 | 0 | 0 | 0 | 0 | 4 |
| % | 0 | 0 | 0 | 0 | 0 | 0 | 0 | 0 | 0 | 0.002287 | 0 | 0 | 0.003087 | 0.003604 | 0.006343 | 0 | 0 | 0 | 0 | 0.008197 |
| Agathobacter | 0 | 0 | 0 | 3 | 0 | 0 | 0 | 0 | 0 | 0 | 0 | 3 | 0 | 0 | 0 | 0 | 3 | 0 | 0 | 0 |
| % | 0 | 0 | 0 | 0.007144 | 0 | 0 | 0 | 0 | 0 | 0 | 0 | 0.005434 | 0 | 0 | 0 | 0 | 0.006605 | 0 | 0 | 0 |
| Curtobacterium | 0 | 0 | 2 | 2 | 0 | 0 | 1 | 0 | 2 | 0 | 0 | 0 | 0 | 0 | 1 | 0 | 0 | 0 | 0 | 0 |
| % | 0 | 0 | 0.005207 | 0.004763 | 0 | 0 | 0.003355 | 0 | 0.005086 | 0 | 0 | 0 | 0 | 0 | 0.002114 | 0 | 0 | 0 | 0 | 0 |
| Chryseobacterium | 3 | 5 | 0 | 0 | 0 | 0 | 0 | 0 | 0 | 0 | 0 | 0 | 0 | 0 | 0 | 0 | 0 | 0 | 0 | 0 |
| % | 0.008965 | 0.021041 | 0 | 0 | 0 | 0 | 0 | 0 | 0 | 0 | 0 | 0 | 0 | 0 | 0 | 0 | 0 | 0 | 0 | 0 |
| Lactonifactor | 0 | 0 | 0 | 0 | 0 | 0 | 0 | 0 | 0 | 0 | 0 | 0 | 2 | 3 | 0 | 0 | 1 | 0 | 0 | 1 |
| % | 0 | 0 | 0 | 0 | 0 | 0 | 0 | 0 | 0 | 0 | 0 | 0 | 0.006174 | 0.010812 | 0 | 0 | 0.002202 | 0 | 0 | 0.002049 |
| Catabacter | 0 | 0 | 0 | 0 | 0 | 0 | 0 | 1 | 1 | 0 | 0 | 0 | 1 | 3 | 0 | 0 | 0 | 0 | 0 | 0 |
| % | 0 | 0 | 0 | 0 | 0 | 0 | 0 | 0.003149 | 0.002543 | 0 | 0 | 0 | 0.003087 | 0.010812 | 0 | 0 | 0 | 0 | 0 | 0 |
| Vagococcus | 0 | 0 | 0 | 0 | 0 | 0 | 0 | 0 | 1 | 0 | 1 | 4 | 0 | 0 | 0 | 0 | 0 | 0 | 0 | 0 |
| % | 0 | 0 | 0 | 0 | 0 | 0 | 0 | 0 | 0.002543 | 0 | 0.001969 | 0.007245 | 0 | 0 | 0 | 0 | 0 | 0 | 0 | 0 |
| Anaerocolumna | 0 | 1 | 2 | 2 | 0 | 0 | 0 | 0 | 0 | 0 | 0 | 0 | 0 | 0 | 0 | 0 | 0 | 0 | 0 | 0 |
| % | 0 | 0.004208 | 0.005207 | 0.004763 | 0 | 0 | 0 | 0 | 0 | 0 | 0 | 0 | 0 | 0 | 0 | 0 | 0 | 0 | 0 | 0 |
| Delftia | 0 | 5 | 0 | 0 | 0 | 0 | 0 | 0 | 0 | 0 | 0 | 0 | 0 | 0 | 0 | 0 | 0 | 0 | 0 | 0 |
| % | 0 | 0.021041 | 0 | 0 | 0 | 0 | 0 | 0 | 0 | 0 | 0 | 0 | 0 | 0 | 0 | 0 | 0 | 0 | 0 | 0 |
| Reyranella | 0 | 0 | 0 | 0 | 1 | 0 | 0 | 0 | 0 | 0 | 0 | 1 | 1 | 0 | 0 | 0 | 1 | 1 | 0 | 0 |
| % | 0 | 0 | 0 | 0 | 0.003651 | 0 | 0 | 0 | 0 | 0 | 0 | 0.001811 | 0.003087 | 0 | 0 | 0 | 0.002202 | 0.002553 | 0 | 0 |
| Enterobacter | 0 | 0 | 0 | 0 | 0 | 5 | 0 | 0 | 0 | 0 | 0 | 0 | 0 | 0 | 0 | 0 | 0 | 0 | 0 | 0 |
| % | 0 | 0 | 0 | 0 | 0 | 0.015479 | 0 | 0 | 0 | 0 | 0 | 0 | 0 | 0 | 0 | 0 | 0 | 0 | 0 | 0 |
| Faecalibacterium | 0 | 0 | 0 | 0 | 0 | 0 | 0 | 0 | 0 | 0 | 1 | 2 | 0 | 0 | 1 | 0 | 0 | 0 | 0 | 1 |
| % | 0 | 0 | 0 | 0 | 0 | 0 | 0 | 0 | 0 | 0 | 0.001969 | 0.003622 | 0 | 0 | 0.002114 | 0 | 0 | 0 | 0 | 0.002049 |
| Bacillus | 0 | 0 | 0 | 0 | 0 | 0 | 0 | 0 | 0 | 0 | 0 | 0 | 0 | 1 | 2 | 1 | 0 | 0 | 0 | 1 |
| % | 0 | 0 | 0 | 0 | 0 | 0 | 0 | 0 | 0 | 0 | 0 | 0 | 0 | 0.003604 | 0.004229 | 0.002749 | 0 | 0 | 0 | 0.002049 |
| Brevundimonas | 1 | 2 | 0 | 0 | 0 | 0 | 0 | 0 | 0 | 0 | 0 | 0 | 0 | 0 | 0 | 0 | 0 | 0 | 0 | 0 |
| % | 0.002988 | 0.008416 | 0 | 0 | 0 | 0 | 0 | 0 | 0 | 0 | 0 | 0 | 0 | 0 | 0 | 0 | 0 | 0 | 0 | 0 |
| Megamonas | 0 | 0 | 0 | 0 | 0 | 0 | 0 | 0 | 1 | 0 | 0 | 0 | 0 | 0 | 2 | 0 | 0 | 0 | 0 | 0 |
| % | 0 | 0 | 0 | 0 | 0 | 0 | 0 | 0 | 0.002543 | 0 | 0 | 0 | 0 | 0 | 0.004229 | 0 | 0 | 0 | 0 | 0 |
| Morganella | 0 | 0 | 1 | 0 | 0 | 0 | 0 | 0 | 0 | 0 | 0 | 0 | 0 | 0 | 0 | 1 | 0 | 0 | 0 | 0 |
| % | 0 | 0 | 0.002603 | 0 | 0 | 0 | 0 | 0 | 0 | 0 | 0 | 0 | 0 | 0 | 0 | 0.002749 | 0 | 0 | 0 | 0 |
| Paraclostridium | 0 | 0 | 0 | 0 | 0 | 0 | 0 | 0 | 0 | 0 | 0 | 0 | 0 | 1 | 0 | 0 | 1 | 0 | 0 | 0 |
| % | 0 | 0 | 0 | 0 | 0 | 0 | 0 | 0 | 0 | 0 | 0 | 0 | 0 | 0.003604 | 0 | 0 | 0.002202 | 0 | 0 | 0 |
| Terrisporobacter | 0 | 0 | 0 | 0 | 0 | 0 | 0 | 0 | 0 | 0 | 0 | 0 | 0 | 0 | 0 | 0 | 0 | 0 | 0 | 2 |
| % | 0 | 0 | 0 | 0 | 0 | 0 | 0 | 0 | 0 | 0 | 0 | 0 | 0 | 0 | 0 | 0 | 0 | 0 | 0 | 0.004098 |
| Anaerotruncus | 0 | 0 | 0 | 0 | 0 | 1 | 0 | 0 | 0 | 0 | 0 | 1 | 0 | 0 | 0 | 0 | 0 | 0 | 0 | 0 |
| % | 0 | 0 | 0 | 0 | 0 | 0.003096 | 0 | 0 | 0 | 0 | 0 | 0.001811 | 0 | 0 | 0 | 0 | 0 | 0 | 0 | 0 |
| Kroppenstedtia | 0 | 0 | 0 | 0 | 0 | 0 | 0 | 0 | 0 | 0 | 0 | 0 | 0 | 0 | 0 | 1 | 0 | 0 | 0 | 0 |
| % | 0 | 0 | 0 | 0 | 0 | 0 | 0 | 0 | 0 | 0 | 0 | 0 | 0 | 0 | 0 | 0.002749 | 0 | 0 | 0 | 0 |
| Methylobacterium | 1 | 0 | 0 | 0 | 0 | 0 | 0 | 0 | 0 | 0 | 0 | 0 | 0 | 0 | 0 | 0 | 0 | 0 | 0 | 0 |
| % | 0.002988 | 0 | 0 | 0 | 0 | 0 | 0 | 0 | 0 | 0 | 0 | 0 | 0 | 0 | 0 | 0 | 0 | 0 | 0 | 0 |
| Aneurinibacillus | 0 | 0 | 0 | 0 | 0 | 0 | 1 | 0 | 0 | 0 | 0 | 0 | 0 | 0 | 0 | 0 | 0 | 0 | 0 | 0 |
| % | 0 | 0 | 0 | 0 | 0 | 0 | 0.003355 | 0 | 0 | 0 | 0 | 0 | 0 | 0 | 0 | 0 | 0 | 0 | 0 | 0 |
| g_[Eubacterium] rectale_incertae_sedis | 0 | 0 | 0 | 0 | 0 | 0 | 0 | 0 | 0 | 0 | 0 | 0 | 0 | 0 | 0 | 1 | 0 | 0 | 0 | 0 |
| % | 0 | 0 | 0 | 0 | 0 | 0 | 0 | 0 | 0 | 0 | 0 | 0 | 0 | 0 | 0 | 0.002749 | 0 | 0 | 0 | 0 |
| Proteus | 0 | 0 | 0 | 0 | 0 | 0 | 0 | 0 | 0 | 0 | 0 | 0 | 0 | 0 | 0 | 0 | 0 | 0 | 0 | 1 |
| % | 0 | 0 | 0 | 0 | 0 | 0 | 0 | 0 | 0 | 0 | 0 | 0 | 0 | 0 | 0 | 0 | 0 | 0 | 0 | 0.002049 |
| Pseudomonas | 0 | 0 | 0 | 0 | 0 | 1 | 0 | 0 | 0 | 0 | 0 | 0 | 0 | 0 | 0 | 0 | 0 | 0 | 0 | 0 |
| % | 0 | 0 | 0 | 0 | 0 | 0.003096 | 0 | 0 | 0 | 0 | 0 | 0 | 0 | 0 | 0 | 0 | 0 | 0 | 0 | 0 |
| Actinomycetospora | 0 | 0 | 0 | 0 | 0 | 0 | 0 | 0 | 0 | 0 | 0 | 0 | 0 | 0 | 0 | 1 | 0 | 0 | 0 | 0 |
| % | 0 | 0 | 0 | 0 | 0 | 0 | 0 | 0 | 0 | 0 | 0 | 0 | 0 | 0 | 0 | 0.002749 | 0 | 0 | 0 | 0 |
| Intestinibacter | 0 | 1 | 0 | 0 | 0 | 0 | 0 | 0 | 0 | 0 | 0 | 0 | 0 | 0 | 0 | 0 | 0 | 0 | 0 | 0 |
| % | 0 | 0.004208 | 0 | 0 | 0 | 0 | 0 | 0 | 0 | 0 | 0 | 0 | 0 | 0 | 0 | 0 | 0 | 0 | 0 | 0 |
| Nubsella | 0 | 1 | 0 | 0 | 0 | 0 | 0 | 0 | 0 | 0 | 0 | 0 | 0 | 0 | 0 | 0 | 0 | 0 | 0 | 0 |
| % | 0 | 0.004208 | 0 | 0 | 0 | 0 | 0 | 0 | 0 | 0 | 0 | 0 | 0 | 0 | 0 | 0 | 0 | 0 | 0 | 0 |
| Eisenbergiella | 0 | 0 | 0 | 0 | 0 | 0 | 0 | 0 | 0 | 0 | 0 | 0 | 0 | 0 | 0 | 0 | 1 | 0 | 0 | 0 |
| % | 0 | 0 | 0 | 0 | 0 | 0 | 0 | 0 | 0 | 0 | 0 | 0 | 0 | 0 | 0 | 0 | 0.002202 | 0 | 0 | 0 |
| Paracoccus | 0 | 0 | 0 | 0 | 0 | 0 | 0 | 0 | 0 | 0 | 0 | 1 | 0 | 0 | 0 | 0 | 0 | 0 | 0 | 0 |
| % | 0 | 0 | 0 | 0 | 0 | 0 | 0 | 0 | 0 | 0 | 0 | 0.001811 | 0 | 0 | 0 | 0 | 0 | 0 | 0 | 0 |
| Microbacterium | 0 | 1 | 0 | 0 | 0 | 0 | 0 | 0 | 0 | 0 | 0 | 0 | 0 | 0 | 0 | 0 | 0 | 0 | 0 | 0 |
| % | 0 | 0.004208 | 0 | 0 | 0 | 0 | 0 | 0 | 0 | 0 | 0 | 0 | 0 | 0 | 0 | 0 | 0 | 0 | 0 | 0 |
| Sanguibacter | 0 | 0 | 0 | 0 | 0 | 0 | 0 | 0 | 0 | 0 | 0 | 0 | 0 | 0 | 1 | 0 | 0 | 0 | 0 | 0 |
| % | 0 | 0 | 0 | 0 | 0 | 0 | 0 | 0 | 0 | 0 | 0 | 0 | 0 | 0 | 0.002114 | 0 | 0 | 0 | 0 | 0 |
| Christensenella | 0 | 0 | 0 | 0 | 0 | 0 | 0 | 0 | 0 | 0 | 0 | 0 | 1 | 0 | 0 | 0 | 0 | 0 | 0 | 0 |
| % | 0 | 0 | 0 | 0 | 0 | 0 | 0 | 0 | 0 | 0 | 0 | 0 | 0.003087 | 0 | 0 | 0 | 0 | 0 | 0 | 0 |
| Collinsella | 0 | 0 | 0 | 0 | 0 | 1 | 0 | 0 | 0 | 0 | 0 | 0 | 0 | 0 | 0 | 0 | 0 | 0 | 0 | 0 |
| % | 0 | 0 | 0 | 0 | 0 | 0.003096 | 0 | 0 | 0 | 0 | 0 | 0 | 0 | 0 | 0 | 0 | 0 | 0 | 0 | 0 |
| Prevotella | 0 | 0 | 0 | 0 | 0 | 0 | 0 | 0 | 0 | 0 | 1 | 0 | 0 | 0 | 0 | 0 | 0 | 0 | 0 | 0 |
| % | 0 | 0 | 0 | 0 | 0 | 0 | 0 | 0 | 0 | 0 | 0.001969 | 0 | 0 | 0 | 0 | 0 | 0 | 0 | 0 | 0 |
| Paeniclostridium | 0 | 0 | 0 | 0 | 0 | 0 | 0 | 0 | 0 | 0 | 0 | 0 | 0 | 0 | 1 | 0 | 0 | 0 | 0 | 0 |
| % | 0 | 0 | 0 | 0 | 0 | 0 | 0 | 0 | 0 | 0 | 0 | 0 | 0 | 0 | 0.002114 | 0 | 0 | 0 | 0 | 0 |
| Novibacillus | 0 | 0 | 0 | 0 | 0 | 0 | 0 | 0 | 0 | 0 | 0 | 0 | 0 | 0 | 1 | 0 | 0 | 0 | 0 | 0 |
| % | 0 | 0 | 0 | 0 | 0 | 0 | 0 | 0 | 0 | 0 | 0 | 0 | 0 | 0 | 0.002114 | 0 | 0 | 0 | 0 | 0 |
| Afipia | 0 | 0 | 0 | 0 | 0 | 0 | 0 | 0 | 0 | 0 | 1 | 0 | 0 | 0 | 0 | 0 | 0 | 0 | 0 | 0 |
| % | 0 | 0 | 0 | 0 | 0 | 0 | 0 | 0 | 0 | 0 | 0.001969 | 0 | 0 | 0 | 0 | 0 | 0 | 0 | 0 | 0 |
| Falcatimonas | 0 | 0 | 0 | 0 | 0 | 0 | 0 | 0 | 0 | 0 | 0 | 1 | 0 | 0 | 0 | 0 | 0 | 0 | 0 | 0 |
| % | 0 | 0 | 0 | 0 | 0 | 0 | 0 | 0 | 0 | 0 | 0 | 0.001811 | 0 | 0 | 0 | 0 | 0 | 0 | 0 | 0 |
| Pediococcus | 0 | 0 | 0 | 0 | 0 | 0 | 0 | 0 | 0 | 0 | 0 | 0 | 0 | 0 | 0 | 0 | 0 | 0 | 0 | 1 |
| % | 0 | 0 | 0 | 0 | 0 | 0 | 0 | 0 | 0 | 0 | 0 | 0 | 0 | 0 | 0 | 0 | 0 | 0 | 0 | 0.002049 |
| Hespellia | 0 | 0 | 0 | 0 | 0 | 0 | 0 | 0 | 0 | 0 | 0 | 0 | 0 | 0 | 0 | 0 | 0 | 0 | 0 | 1 |
| % | 0 | 0 | 0 | 0 | 0 | 0 | 0 | 0 | 0 | 0 | 0 | 0 | 0 | 0 | 0 | 0 | 0 | 0 | 0 | 0.002049 |
| Lysinibacillus | 0 | 0 | 0 | 0 | 0 | 0 | 0 | 0 | 0 | 0 | 0 | 0 | 0 | 0 | 1 | 0 | 0 | 0 | 0 | 0 |
| % | 0 | 0 | 0 | 0 | 0 | 0 | 0 | 0 | 0 | 0 | 0 | 0 | 0 | 0 | 0.002114 | 0 | 0 | 0 | 0 | 0 |
| Facklamia | 0 | 0 | 0 | 0 | 0 | 0 | 0 | 0 | 0 | 0 | 0 | 0 | 0 | 0 | 1 | 0 | 0 | 0 | 0 | 0 |
| % | 0 | 0 | 0 | 0 | 0 | 0 | 0 | 0 | 0 | 0 | 0 | 0 | 0 | 0 | 0.002114 | 0 | 0 | 0 | 0 | 0 |
| Barrientosiimonas | 0 | 0 | 0 | 0 | 0 | 0 | 0 | 0 | 0 | 0 | 0 | 0 | 0 | 0 | 0 | 0 | 0 | 1 | 0 | 0 |
| % | 0 | 0 | 0 | 0 | 0 | 0 | 0 | 0 | 0 | 0 | 0 | 0 | 0 | 0 | 0 | 0 | 0 | 0.002553 | 0 | 0 |
| Saccharomonospora | 0 | 0 | 0 | 0 | 0 | 0 | 0 | 0 | 0 | 0 | 0 | 0 | 0 | 0 | 1 | 0 | 0 | 0 | 0 | 0 |
| % | 0 | 0 | 0 | 0 | 0 | 0 | 0 | 0 | 0 | 0 | 0 | 0 | 0 | 0 | 0.002114 | 0 | 0 | 0 | 0 | 0 |
| Massilia | 0 | 0 | 0 | 0 | 0 | 1 | 0 | 0 | 0 | 0 | 0 | 0 | 0 | 0 | 0 | 0 | 0 | 0 | 0 | 0 |
| % | 0 | 0 | 0 | 0 | 0 | 0.003096 | 0 | 0 | 0 | 0 | 0 | 0 | 0 | 0 | 0 | 0 | 0 | 0 | 0 | 0 |
| Phascolarctobacterium | 0 | 0 | 1 | 0 | 0 | 0 | 0 | 0 | 0 | 0 | 0 | 0 | 0 | 0 | 0 | 0 | 0 | 0 | 0 | 0 |
| % | 0 | 0 | 0.002603 | 0 | 0 | 0 | 0 | 0 | 0 | 0 | 0 | 0 | 0 | 0 | 0 | 0 | 0 | 0 | 0 | 0 |
| Lachnotalea | 0 | 0 | 0 | 0 | 0 | 0 | 0 | 0 | 1 | 0 | 0 | 0 | 0 | 0 | 0 | 0 | 0 | 0 | 0 | 0 |
| % | 0 | 0 | 0 | 0 | 0 | 0 | 0 | 0 | 0.002543 | 0 | 0 | 0 | 0 | 0 | 0 | 0 | 0 | 0 | 0 | 0 |
| Butyricimonas | 0 | 0 | 0 | 0 | 0 | 0 | 1 | 0 | 0 | 0 | 0 | 0 | 0 | 0 | 0 | 0 | 0 | 0 | 0 | 0 |
| % | 0 | 0 | 0 | 0 | 0 | 0 | 0.003355 | 0 | 0 | 0 | 0 | 0 | 0 | 0 | 0 | 0 | 0 | 0 | 0 | 0 |
| Rhizobium | 0 | 0 | 0 | 1 | 0 | 0 | 0 | 0 | 0 | 0 | 0 | 0 | 0 | 0 | 0 | 0 | 0 | 0 | 0 | 0 |
| % | 0 | 0 | 0 | 0.002381 | 0 | 0 | 0 | 0 | 0 | 0 | 0 | 0 | 0 | 0 | 0 | 0 | 0 | 0 | 0 | 0 |
| Total count | 33462 | 23763 | 38413 | 41991 | 27388 | 32301 | 29805 | 31752 | 39320 | 43726 | 50797 | 55212 | 32395 | 27746 | 47298 | 36379 | 45420 | 39174 | 30346 | 48799 |

C-numbered, N-numbered, and P-numbered samples are obtained from controls, Nx (5/6 nephrectomy) groups, and Nx + PAR (5/6 nephrectomy + 5% paramylon treatment) groups, respectively.
