# Supplementary material for: Renoprotective effects of paramylon, a β-1,3-D-Glucan isolated from Euglena gracilis Z in a rodent model of chronic kidney disease
Source: PLoS One. 2020 Aug 7;15(8):e0237086. doi: 10.1371/journal.pone.0237086 (PMC7413521; doi:10.1371/journal.pone.0237086)
Supplement: S7 Table — (DOCX) [file pone.0237086.s008.docx]

| **Anionic compounds** | **Previous reports as uremic toxin** | **References** |
| --- | --- | --- |
| cis-aconitic acid  citric acid  malic acid | ↑in CKD mice  ↑in CKD mice  ↑in CKD mice | [1]  [1]  [1] |
| homovanillic acid  isocitric acid | ↑in CKD mice and ↑in CKD patients  ↑in CKD mice and ↑in CKD patients | [1], [2]  [1], [2] |
| 5-oxoproline | unknown |  |
| **Cationic compounds** | **Previous reports as uremic toxin** | **References** |
| 1-methylhistidine  4-guanidinobutyric acid  citrulline  N, N-dimethylglycine  N6, N6, N6-trimethyllysine  pipecolic acid  sarcosine  trigonelline | ↑in CKD mice  ↑in CKD mice  ↑in CKD mice  ↑in CKD mice  ↑in CKD mice  ↑in CKD mice  ↑in CKD mice  ↑in CKD mice | [1]  [1]  [1]  [1]  [1]  [1]  [1]  [1] |
| ADMA  SDMA | ↑in CKD mice and ↑in CKD patients  ↑in CKD mice and ↑in CKD patients | [1], [3]  [1], [3] |
| Urea | ↑in CKD mice and ↑in CKD patients | [1], [4] |
| 1-methyladenosine | ↑in CKD patients | [5] |
| 1-methylnicotinamide | ↑in CKD patients | [3] |
| cystathionine | ↑in hemodialysis patients | [6] |
| homocitrulline | ↑in CKD patients | [7] |
| 5-hydroxylysine  argininosuccinic acid  N6-methyllysine  N8-acetylspermidine  O-acetylhomoserine  galactosylhydroxylysine | unknown  unknown  unknown  unknown  unknown  unknown |  |

CKD, chronic kidney disease; ADMA, asymmetric dimethylarginine; SDMA, [symmetric dimethylarginine](http://onlinelibrary.wiley.com/doi/10.1111/jvim.12835/abstract).

**Supplementary references**

1. Mishima E, Fukuda S, Mukawa C, Yuri A, Kanemitsu Y, Matsumoto Y, et al. Evaluation of the impact of gut microbiota on uremic solute accumulation by a CE-TOFMS-based metabolomics approach. Kidney Int. 2017;92(3):634-45. Epub 2017/04/12. doi: 10.1016/j.kint.2017.02.011. PubMed PMID: 28396122.

2. Rhee EP, Souza A, Farrell L, Pollak MR, Lewis GD, Steele DJ, et al. Metabolite profiling identifies markers of uremia. J Am Soc Nephrol. 2010;21(6):1041-51. Epub 2010/04/10. doi: 10.1681/asn.2009111132. PubMed PMID: 20378825; PubMed Central PMCID: PMCPMC2900954.

3. Duranton F, Cohen G, De Smet R, Rodriguez M, Jankowski J, Vanholder R, et al. Normal and pathologic concentrations of uremic toxins. J Am Soc Nephrol. 2012;23(7):1258-70. Epub 2012/05/26. doi: 10.1681/asn.2011121175. PubMed PMID: 22626821; PubMed Central PMCID: PMCPMC3380651.

4. Wengle B, Hellstrom K. Volatile phenols in serum of uraemic patients. Clin Sci. 1972;43(4):493-8. Epub 1972/10/01. doi: 10.1042/cs0430493. PubMed PMID: 4653588.

5. Niwa T, Takeda N, Yoshizumi H. RNA metabolism in uremic patients: accumulation of modified ribonucleosides in uremic serum. Technical note. Kidney Int. 1998;53(6):1801-6. Epub 1998/06/02. doi: 10.1046/j.1523-1755.1998.00944.x. PubMed PMID: 9607216.

6. Perna AF, Di Nunzio A, Amoresano A, Pane F, Fontanarosa C, Pucci P, et al. Divergent behavior of hydrogen sulfide pools and of the sulfur metabolite lanthionine, a novel uremic toxin, in dialysis patients. Biochimie. 2016;126:97-107. Epub 2016/05/01. doi: 10.1016/j.biochi.2016.04.018. PubMed PMID: 27129884.

7. Kraus LM, Kraus AP, Jr. The search for the uremic toxin: the case for carbamoylation of amino acids and proteins. Wien Klin Wochenschr. 1998;110(15):521-30. Epub 1998/10/23. PubMed PMID: 9782570.
