# Supplementary material for: Renoprotective effects of paramylon, a β-1,3-D-Glucan isolated from Euglena gracilis Z in a rodent model of chronic kidney disease
Source: PLoS One. 2020 Aug 7;15(8):e0237086. doi: 10.1371/journal.pone.0237086 (PMC7413521; doi:10.1371/journal.pone.0237086)
Supplement: S8 Table — (DOCX) [file pone.0237086.s009.docx]

Body weight (g) at 8wk.

| Control (n=4) | Nx (n=8) | Nx + PAR (n=8) |
| --- | --- | --- |
| 302 | 262 | 285 |
| 315 | 274 | 277 |
| 259 | 306 | 261 |
| 272 | 224 | 278 |
|  | 279 | 261 |
|  | 253 | 274 |
|  | 232 | 279 |
|  | 275 | 249 |

Diet intake (g/day).

| Control (n=4) | Nx (n=8) | Nx + PAR (n=8) |
| --- | --- | --- |
| 14 | 7 | 13 |
| 15 | 11 | 15 |
| 20 | 15 | 14 |
| 21 | 6 | 15 |
|  | 12 | 15 |
|  | 14 | 17 |
|  | 17 | 18 |
|  | 6 | 13 |

Water intake (g/day).

| Control (n=4) | Nx (n=8) | Nx + PAR (n=8) |
| --- | --- | --- |
| 14 | 31 | 47 |
| 15 | 35 | 33 |
| 21 | 41 | 27 |
| 22 | 30 | 31 |
|  | 49 | 35 |
|  | 48 | 59 |
|  | 59 | 60 |
|  | 2 | 55 |

Serum UN (mg/dL).

| Control (n=4) | Nx (n=8) | Nx + PAR (n=8) |
| --- | --- | --- |
| 20 | 74.6 | 51.8 |
| 20 | 60.4 | 44.6 |
| 16.5 | 35.4 | 37.4 |
| 19.5 | 128.4 | 34.8 |
|  | 52.5 | 41.8 |
|  | 50.2 | 56.7 |
|  | 66.5 | 36.9 |
|  | 48.8 | 47 |

Serum Cr (mg/dL).

| Control (n=4) | Nx (n=8) | Nx + PAR (n=8) |
| --- | --- | --- |
| 0.36 | 1.34 | 0.87 |
| 0.37 | 1.07 | 0.64 |
| 0.25 | 0.49 | 0.63 |
| 0.35 | 3.19 | 0.62 |
|  | 0.78 | 0.66 |
|  | 0.89 | 1.09 |
|  | 1.2 | 0.59 |
|  | 0.86 | 0.73 |

Ccr (ml/min/100g BW).

| Control (n=4) | Nx (n=8) | Nx + PAR (n=8) |
| --- | --- | --- |
| 72.61531 | 16.49047 | 26.21496 |
| 76.20402 | 17.27877 | 32.65723 |
| 96.2281 | 47.38738 | 40.33071 |
| 77.83883 | 4.25363 | 36.64301 |
|  | 32.87311 | 39.29659 |
|  | 25.96329 | 18.02925 |
|  | 12.84814 | 33.41231 |
|  | 23.41844 | 27.64835 |

Serum total cholesterol (mg/dL).

| Control (n=4) | Nx (n=8) | Nx + PAR (n=8) |
| --- | --- | --- |
| 96 | 332 | 252 |
| 92 | 184 | 136 |
| 66 | 111 | 112 |
| 68 | 370 | 120 |
|  | 180 | 203 |
|  | 208 | 152 |
|  | 287 | 127 |
|  | 141 | 128 |

Urinary protein (mg/mg Cr) at 8wk.

| Control (n=4) | Nx (n=8) | Nx + PAR (n=8) |
| --- | --- | --- |
| 4 | 215.9091 | 123.4211 |
| 4.444444 | 88.28571 | 52.72727 |
| 3.888889 | 32.40741 | 13.33333 |
| 4.705883 | 466.6667 | 24.5 |
|  | 113.5484 | 136.875 |
|  | 125 | 91.71429 |
|  | 184.5161 | 29.48718 |
|  | 53.61111 | 60.71429 |
