# Supplementary material for: Renoprotective effects of paramylon, a β-1,3-D-Glucan isolated from Euglena gracilis Z in a rodent model of chronic kidney disease
Source: PLoS One. 2020 Aug 7;15(8):e0237086. doi: 10.1371/journal.pone.0237086 (PMC7413521; doi:10.1371/journal.pone.0237086)
Supplement: S9 Table — (DOCX) [file pone.0237086.s010.docx]

|  | Control | | | Nx | | | Nx + PAR | | |
| --- | --- | --- | --- | --- | --- | --- | --- | --- | --- |
|  | Mean SEM N | | | Mean SEM N | | | Mean SEM N | | |
| day7 | 186 | 6.646 | 4 | 162.9 | 2.133 | 8 | 162.1 | 0.8332 | 8 |
| day14 | 207.3 | 8.045 | 4 | 189 | 3.065 | 8 | 191.8 | 1.13 | 8 |
| day21 | 231.3 | 9.911 | 4 | 210.8 | 4.35 | 8 | 219 | 1.982 | 8 |
| day28 | 243 | 11.13 | 4 | 225.9 | 4.658 | 8 | 233.9 | 3.226 | 8 |
| day35 | 259 | 11.99 | 4 | 239.5 | 5.359 | 8 | 248.3 | 3.629 | 8 |
| day42 | 271 | 11.97 | 4 | 249.4 | 6.225 | 8 | 258.1 | 2.961 | 8 |
| day49 | 284.3 | 13.43 | 4 | 262 | 7.693 | 8 | 270.6 | 3.201 | 8 |
| day56 | 294.3 | 13 | 4 | 267.8 | 8.353 | 8 | 276.4 | 3.257 | 8 |
| day63 | 303.5 | 13.55 | 4 | 274.5 | 9.686 | 8 | 285.4 | 3.937 | 8 |
| day66 | 287 | 12.97 | 4 | 263.1 | 9.404 | 8 | 270.5 | 4.301 | 8 |

Body weight (g).

Urinary protein (mg/mg Cr).

|  | Control | | | Nx | | | Nx + PAR | | |
| --- | --- | --- | --- | --- | --- | --- | --- | --- | --- |
|  | Mean SEM N | | | Mean SEM N | | | Mean SEM N | | |
| 0 | 6.144 | 0.2113 | 4 | 9.364 | 1.137 | 8 | 7.976 | 0.9578 | 8 |
| 2week | 7.275 | 0.6998 | 4 | 25.22 | 10.46 | 8 | 9.851 | 1.327 | 8 |
| 4week | 6.151 | 1.288 | 4 | 50.38 | 13.26 | 8 | 31.48 | 9.173 | 8 |
| 6week | 5.185 | 0.7651 | 4 | 64.04 | 17.65 | 8 | 32.12 | 7.001 | 8 |
| 8week | 4.26 | 0.1911 | 4 | 160 | 48.9 | 8 | 66.6 | 16.37 | 8 |
