# Supplementary material for: Renoprotective effects of paramylon, a β-1,3-D-Glucan isolated from Euglena gracilis Z in a rodent model of chronic kidney disease
Source: PLoS One. 2020 Aug 7;15(8):e0237086. doi: 10.1371/journal.pone.0237086 (PMC7413521; doi:10.1371/journal.pone.0237086)
Supplement: S10 Table — (DOCX) [file pone.0237086.s011.docx]

Glomerular sclerotic score.

| Control (n=4) | Nx (n=8) | Nx + PAR (n=8) |
| --- | --- | --- |
| 0 | 2.51 | 1.57 |
| 0 | 0.92 | 0.81 |
| 0 | 0.38 | 0.47 |
| 0 | 3.52 | 0.19 |
|  | 1.47 | 1.22 |
|  | 1.49 | 1.49 |
|  | 2.41 | 0.48 |
|  | 1.44 | 0.85 |

Tubular injury score.

| Control (n=4) | Nx (n=8) | Nx + PAR (n=8) |
| --- | --- | --- |
| 0 | 3.5 | 2.45 |
| 0 | 2.64 | 0.58 |
| 0 | 1.25 | 0.85 |
| 0 | 4 | 0.075 |
|  | 1.95 | 1.1 |
|  | 3.05 | 1.53 |
|  | 3.78 | 0.78 |
|  | 2.6 | 1 |

Glomerular area (µm^2^).

| Control (n=4) | Nx (n=8) | Nx + PAR (n=8) |
| --- | --- | --- |
| 6315 | 10745 | 8894 |
| 6544 | 10320 | 12049 |
| 5753 | 11789 | 9429 |
| 6539 | 10216 | 9504 |
|  | 10704 | 9497 |
|  | 10110 | 9663 |
|  | 9100 | 8852 |
|  | 11095 | 8684 |

The number of foot processes per micrometer of glomerular basement membrane.

| Control (n=3) | Nx (n=3) | Nx + PAR (n=3) |
| --- | --- | --- |
| 2.2 | 0.77 | 2 |
| 2.3 | 1.5 | 2 |
| 2.2 | 1.3 | 1.7 |
