# Supplementary material for: Renoprotective effects of paramylon, a β-1,3-D-Glucan isolated from Euglena gracilis Z in a rodent model of chronic kidney disease
Source: PLoS One. 2020 Aug 7;15(8):e0237086. doi: 10.1371/journal.pone.0237086 (PMC7413521; doi:10.1371/journal.pone.0237086)
Supplement: S11 Table — (DOCX) [file pone.0237086.s012.docx]

Fibrotic area (%).

| Control (n=4) | Nx (n=8) | Nx + PAR (n=8) |
| --- | --- | --- |
| 1.21 | 5.37 | 1.75 |
| 1.19 | 2.33 | 2.2 |
| 0.69 | 2.41 | 1.34 |
| 0.84 | 13.4 | 0.87 |
|  | 4.44 | 1.72 |
|  | 8.72 | 7.51 |
|  | 14.6 | 3.33 |
|  | 9.03 | 7.37 |

α-SMA area (%).

| Control (n=4) | Nx (n=8) | Nx + PAR (n=8) |
| --- | --- | --- |
| 0.183 | 0.563 | 0.538 |
| 0.2 | 0.486 | 0.102 |
| 0.096 | 0.363 | 0.201 |
| 0.067 | 0.364 | 0.117 |
|  | 0.666 | 0.37 |
|  | 0.622 | 0.56 |
|  | 0.913 | 0.799 |
|  | 0.931 | 0.57 |

The number of tubulointerstitial ED-1 positive cells.

| Control (n=4) | Nx (n=8) | Nx + PAR (n=8) |
| --- | --- | --- |
| 5.2 | 46.3 | 47.6 |
| 7.5 | 31.15 | 22.4 |
| 8.95 | 23.25 | 18.7 |
| 6.7 | 79.1 | 19.65 |
|  | 47.15 | 50.8 |
|  | 76.9 | 75.25 |
|  | 66.6 | 62.95 |
|  | 54.8 | 26.1 |

The number of tubulointerstitial CD3 positive cells.

| Control (n=4) | Nx (n=8) | Nx + PAR (n=8) |
| --- | --- | --- |
| 6.55 | 48.35 | 51.55 |
| 7.7 | 42.15 | 27.85 |
| 6.4 | 29.3 | 16.45 |
| 3.25 | 59.3 | 15.05 |
|  | 40.75 | 38 |
|  | 41.65 | 50.2 |
|  | 39.45 | 42.2 |
|  | 30 | 23.75 |

The number of tubulointerstitial PCNA positive cells.

| Control (n=4) | Nx (n=8) | Nx + PAR (n=8) |
| --- | --- | --- |
| 29 | 90.5 | 62.95 |
| 20.15 | 102.3 | 38.75 |
| 3.2 | 47.25 | 22.9 |
| 6.15 | 99.7 | 19.35 |
|  | 44.3 | 38.9 |
|  | 37.35 | 61.65 |
|  | 101.1 | 29.25 |
|  | 57.25 | 14.6 |
