# Supplementary material for: Renoprotective effects of paramylon, a β-1,3-D-Glucan isolated from Euglena gracilis Z in a rodent model of chronic kidney disease
Source: PLoS One. 2020 Aug 7;15(8):e0237086. doi: 10.1371/journal.pone.0237086 (PMC7413521; doi:10.1371/journal.pone.0237086)
Supplement: S12 Table — (DOCX) [file pone.0237086.s013.docx]

*Ccl2*

| Control (n=4) | Nx (n=8) | Nx + PAR (n=8) |
| --- | --- | --- |
| 1 | 19.43 | 5.66 |
| 0.97 | 7.01 | 2.77 |
| 2.51 | 2.69 | 1.05 |
| 0.92 | 34.06 | 1.01 |
|  | 7.01 | 3.61 |
|  | 5.1 | 7.84 |
|  | 9.85 | 3.01 |
|  | 3.32 | 2.33 |

*Tnfa*

| Control (n=4) | Nx (n=8) | Nx + PAR (n=8) |
| --- | --- | --- |
| 1 | 5.43 | 3.18 |
| 1.51 | 2.46 | 2.58 |
| 1.83 | 2.25 | 1.64 |
| 1.02 | 7.57 | 1.04 |
|  | 4.26 | 2.64 |
|  | 2.64 | 4.63 |
|  | 3.61 | 2.79 |
|  | 2.14 | 2.51 |

*Serpine1*

| Control (n=4) | Nx (n=8) | Nx + PAR (n=8) |
| --- | --- | --- |
| 1 | 34.54 | 10.56 |
| 1.31 | 8.22 | 10.63 |
| 2.11 | 3.76 | 2.03 |
| 4.17 | 38.59 | 3.27 |
|  | 21.71 | 7.89 |
|  | 9.13 | 16.11 |
|  | 34.78 | 4.44 |
|  | 4.26 | 10.7 |

*Il-1b*

| Control (n=4) | Nx (n=8) | Nx + PAR (n=8) |
| --- | --- | --- |
| 1 | 10.63 | 2.64 |
| 1.49 | 4.5 | 2.57 |
| 3.2 | 3.01 | 0.8 |
| 3.2 | 8.82 | 0.99 |
|  | 3.46 | 1.37 |
|  | 1.43 | 3.43 |
|  | 3.66 | 1.26 |
|  | 1.38 | 0.9 |

*Tgfb1*

| Control (n=4) | Nx (n=8) | Nx + PAR (n=8) |
| --- | --- | --- |
| 1 | 4.44 | 1.99 |
| 0.7 | 1.27 | 1.23 |
| 0.7 | 0.93 | 0.93 |
| 0.82 | 5.94 | 0.99 |
|  | 2.91 | 1.36 |
|  | 1.69 | 2.97 |
|  | 3.97 | 1.37 |
|  | 1.16 | 1.6 |

*Col1a1*

| Control (n=4) | Nx (n=8) | Nx + PAR (n=8) |
| --- | --- | --- |
| 1 | 18.77 | 6.63 |
| 0.91 | 3.84 | 3.29 |
| 1.01 | 2.25 | 2.57 |
| 1.19 | 13.27 | 2.68 |
|  | 10.7 | 2.91 |
|  | 4.86 | 11 |
|  | 15.89 | 3.76 |
|  | 2.22 | 3.68 |

*Mmp9*

| Control (n=4) | Nx (n=8) | Nx + PAR (n=8) |
| --- | --- | --- |
| 1 | 0.48 | 0.26 |
| 0.88 | 0.31 | 0.83 |
| 1.67 | 0.87 | 0.62 |
| 1.28 | 0.14 | 0.47 |
|  | 0.73 | 0.6 |
|  | 0.05 | 0.85 |
|  | 0.22 | 0.25 |
|  | 0.21 | 0.31 |

*Ifng*

| Control (n=4) | Nx (n=8) | Nx + PAR (n=8) |
| --- | --- | --- |
| 1 | 4.41 | 2.75 |
| 1.28 | 2.68 | 3.18 |
| 2.89 | 3.53 | 1.42 |
| 1.42 | 5.31 | 1.25 |
|  | 2.95 | 3.51 |
|  | 3.53 | 4.35 |
|  | 4 | 5.17 |
|  | 1.89 | 1.96 |

*Nos2*

| Control (n=4) | Nx (n=8) | Nx + PAR (n=8) |
| --- | --- | --- |
| 1 | 19.97 | 2.16 |
| 0.68 | 7.84 | 1.11 |
| 0.12 | 0.65 | 0.44 |
| 0.32 | 41.36 | 0.42 |
|  | 2.79 | 3.53 |
|  | 2.41 | 23.92 |
|  | 11.96 | 3.71 |
|  | 3.81 | 2.23 |

*Il10*

| Control (n=4) | Nx (n=8) | Nx + PAR (n=8) |
| --- | --- | --- |
| 1 | 6.68 | 2.22 |
| 1.11 | 1.89 | 0.37 |
| 2.03 | 0.69 | 0.54 |
| 1.97 | 11.96 | 0.59 |
|  | 3.63 | 0.71 |
|  | 1.09 | 3.34 |
|  | 4.5 | 1.75 |
|  | 0.72 | 0.36 |

*Il4*

| Control (n=4) | Nx (n=8) | Nx + PAR (n=8) |
| --- | --- | --- |
| 1 | 2.81 | 1.28 |
| 0.73 | 0.19 | 1.26 |
| 1.3 | 1.04 | 0.54 |
| 0.86 | 6.68 | 0.8 |
|  | 1.61 | 1 |
|  | 1.25 | 0.7 |
|  | 1.88 | 0.62 |
|  | 0.97 | 0.96 |
