# Supplementary material for: Renoprotective effects of paramylon, a β-1,3-D-Glucan isolated from Euglena gracilis Z in a rodent model of chronic kidney disease
Source: PLoS One. 2020 Aug 7;15(8):e0237086. doi: 10.1371/journal.pone.0237086 (PMC7413521; doi:10.1371/journal.pone.0237086)
Supplement: S13 Table — (DOCX) [file pone.0237086.s014.docx]

cis-Aconitic acid

| Control (n=4) | Nx (n=7) | Nx + PAR (n=8) |
| --- | --- | --- |
| 17 | 37 | 30 |
| 15 | 35 | 24 |
| 16 | 43 | 23 |
| 17 | 28 | 29 |
|  | 31 | 27 |
|  | 34 | 30 |
|  | 29 | 25 |
|  |  | 25 |

Citric acid

| Control (n=4) | Nx (n=7) | Nx + PAR (n=8) |
| --- | --- | --- |
| 333 | 877 | 627 |
| 309 | 775 | 466 |
| 260 | 1026 | 524 |
| 340 | 520 | 479 |
|  | 601 | 603 |
|  | 710 | 698 |
|  | 648 | 477 |
|  |  | 545 |

Isocitric acid

| Control (n=4) | Nx (n=7) | Nx + PAR (n=8) |
| --- | --- | --- |
| 29 | 62 | 48 |
| 28 | 56 | 37 |
| 23 | 77 | 42 |
| 26 | 43 | 36 |
|  | 41 | 46 |
|  | 50 | 53 |
|  | 49 | 36 |
|  |  | 36 |

Maric acid

| Control (n=4) | Nx (n=7) | Nx + PAR (n=8) |
| --- | --- | --- |
| 143 | 216 | 136 |
| 88 | 145 | 37 |
| 47 | 203 | 103 |
| 73 | 106 | 109 |
|  | 122 | 130 |
|  | 150 | 100 |
|  | 152 | 88 |
|  |  | 118 |

Fumaric acid

| Control (n=2) | Nx (n=7) | Nx + PAR (n=6) |
| --- | --- | --- |
| 21 | 35 | 22 |
| 14 | 28 | 18 |
|  | 32 | 19 |
|  | 11 | 17 |
|  | 20 | 15 |
|  | 21 | 16 |
|  | 23 |  |

Succinic acid

| Control (n=4) | Nx (n=7) | Nx + PAR (n=8) |
| --- | --- | --- |
| 50 | 72 | 40 |
| 46 | 46 | 23 |
| 34 | 61 | 23 |
| 34 | 29 | 24 |
|  | 37 | 32 |
|  | 44 | 27 |
|  | 44 | 23 |
|  |  | 27 |
