# Supplementary material for: Renoprotective effects of paramylon, a β-1,3-D-Glucan isolated from Euglena gracilis Z in a rodent model of chronic kidney disease
Source: PLoS One. 2020 Aug 7;15(8):e0237086. doi: 10.1371/journal.pone.0237086 (PMC7413521; doi:10.1371/journal.pone.0237086)
Supplement: Supplementary file 14 [file pone.0237086.s004.docx]

ID	HMT DB			Relative Area					
	Compound name	KEGG ID	HMDB ID	Control		Nx		Nx + PAR	
				Mean	S.D.	Mean	S.D.	Mean	S.D.
C_0136	1-Methyladenosine	C02494	HMDB03331	7.6E-05	1.1E-05	1.4E-04	1.4E-05	1.1E-04	1.4E-05
C_0087	1-Methylhistidine<br>3-Methylhistidine	C01152	HMDB00001, HMDB00479	9.1E-03	8.2E-04	1.8E-02	3.1E-03	1.4E-02	1.7E-03
C_0055	1-Methylnicotinamide	C02918	HMDB00699	8.8E-04	1.7E-04	2.2E-03	6.1E-04	1.6E-03	2.8E-04
C_0060	1*H* -Imidazole-4-propionic acid			1.2E-04	6.4E-05	2.7E-04	1.3E-04	1.7E-04	8.2E-05
C_0117	2'-Deoxycytidine	C00881	HMDB00014	9.5E-03	6.8E-04	8.0E-03	3.4E-04	7.9E-03	6.2E-04
C_0118	2'-Deoxyuridine	C00526	HMDB00012	2.3E-04	3.1E-05	2.5E-04	2.6E-05	2.1E-04	3.4E-05
C_0018	2-Aminoisobutyric acid<br>2-Aminobutyric acid	C03665, C02261, C02356	HMDB01906, HMDB00452	5.5E-03	2.4E-03	5.4E-03	2.3E-03	5.2E-03	1.1E-03
A_0025	2-Hydroxy-4-methylvaleric acid	C03264	HMDB00624	3.7E-04	7.0E-05	7.8E-04	4.6E-04	3.6E-04	4.1E-05
A_0010	2-Hydroxybutyric acid	C05984	HMDB00008	2.2E-03	6.8E-04	2.2E-03	5.9E-04	2.3E-03	5.3E-04
A_0035	2-Hydroxyglutaric acid	C02630, C01087, C03196	HMDB00606, HMDB00694	N.A.	N.A.	4.2E-04	9.1E-05	3.5E-04	N.A.
A_0008	2-Hydroxyisobutyric acid		HMDB00729	7.7E-04	1.6E-04	8.4E-04	1.9E-04	1.0E-03	1.9E-04
A_0018	2-Hydroxyvaleric acid		HMDB01863	7.7E-04	6.1E-05	2.0E-03	1.5E-03	9.4E-04	1.8E-04
A_0033	2-Oxoglutaric acid	C00026	HMDB00208	6.1E-03	1.0E-03	6.2E-03	1.0E-03	4.5E-03	8.4E-04
A_0014	2-Oxoisovaleric acid	C00141	HMDB00019	2.7E-03	3.1E-04	2.1E-03	2.8E-04	2.1E-03	3.4E-04
C_0017	3-Aminobutyric acid			4.2E-04	7.3E-05	5.0E-04	1.1E-04	5.8E-04	7.0E-05
A_0039	3-Hydroxy-3-methylglutaric acid	C03761		3.6E-04	N.A.	4.0E-04	1.0E-04	3.1E-04	8.7E-05
A_0009	3-Hydroxybutyric acid	C01089, C03197	HMDB00011, HMDB00357, HMDB00442	2.5E-02	5.6E-03	3.1E-02	1.0E-02	4.8E-02	1.6E-02
A_0070	3-Indoxylsulfuric acid		HMDB00682	2.9E-03	1.1E-03	9.7E-03	9.4E-03	5.1E-03	9.0E-04
A_0058	3-Phosphoglyceric acid	C00197	HMDB00807	N.A.	N.A.	2.4E-04	N.A.	N.A.	N.A.
C_0076	4-(β-Acetylaminoethyl)imidazole	C05135		N.A.	N.A.	5.2E-05	2.3E-05	N.A.	N.A.
A_0032	4-Acetamidobutanoic acid	C02946	HMDB03681	N.A.	N.A.	2.7E-04	1.3E-04	1.9E-04	4.2E-05
C_0064	4-Guanidinobutyric acid	C01035	HMDB03464	1.8E-04	9.0E-05	3.8E-03	1.8E-03	1.6E-03	1.0E-03
A_0021	4-Methyl-2-oxovaleric acid<br>3-Methyl-2-oxovaleric acid	C00233, C00671, C03465	HMDB00695, HMDB00491	2.2E-02	3.8E-03	1.7E-02	2.5E-03	1.8E-02	4.8E-03
A_0056	4-Pyridoxic acid	C00847	HMDB00017	N.A.	N.A.	2.9E-04	1.8E-04	N.A.	N.A.
C_0082	5-Hydroxylysine	C16741	HMDB00450	2.6E-04	4.4E-05	5.1E-04	8.7E-05	3.7E-04	4.1E-05
C_0125	5-Methyl-2'-deoxycytidine	C03592	HMDB02224	2.2E-04	3.2E-05	3.4E-04	1.2E-04	2.6E-04	2.9E-05
C_0039	5-Methylcytosine	C02376	HMDB02894	N.A.	N.A.	1.2E-04	N.A.	N.A.	N.A.
A_0020	5-Oxoproline	C01879	HMDB00267	8.6E-04	4.0E-05	1.3E-03	2.9E-04	9.8E-04	1.6E-04
C_0122	7,8-Dihydrobiopterin	C02953	HMDB00038	N.A.	N.A.	2.5E-05	1.8E-06	N.A.	N.A.
C_0084	7-Methylguanine	C02242	HMDB00897	N.A.	N.A.	1.4E-04	6.0E-05	9.0E-05	1.1E-05
C_0015	Acetoacetamide	C11106		N.A.	N.A.	1.2E-04	3.5E-05	N.A.	N.A.
A_0006	Acetoacetic acid	C00164	HMDB00060	4.1E-04	1.2E-04	5.8E-04	2.2E-04	1.2E-03	5.3E-04
A_0034	Adipic acid	C06104	HMDB00448	N.A.	N.A.	4.8E-04	1.9E-04	7.5E-04	N.A.
C_0105	ADMA	C03626	HMDB01539	4.3E-04	6.7E-05	5.3E-04	7.0E-05	4.1E-04	5.6E-05
C_0012	Ala	C00041, C00133, C01401	HMDB00161, HMDB01310	1.3E-01	2.2E-02	1.4E-01	2.5E-02	1.1E-01	1.7E-02
A_0052	Allantoic acid	C00499	HMDB01209	N.A.	N.A.	3.3E-04	3.2E-04	1.5E-04	3.2E-05
C_0035	Anserine_divalent	C01262	HMDB00194	7.7E-04	2.8E-04	6.2E-04	1.6E-04	1.4E-03	7.2E-04
C_0089	Arg	C00062, C00792	HMDB00517, HMDB03416	6.6E-02	1.0E-02	6.9E-02	1.4E-02	5.2E-02	6.2E-03
C_0139	Argininosuccinic acid	C03406	HMDB00052	1.4E-04	5.0E-05	6.3E-04	2.6E-04	3.1E-04	8.7E-05
C_0049	Asn	C00152, C01905, C16438	HMDB00168	1.3E-02	2.0E-03	1.2E-02	2.5E-03	1.0E-02	1.5E-03
C_0051	Asp	C00049, C00402, C16433	HMDB00191, HMDB06483	1.7E-02	2.4E-03	1.5E-02	5.0E-03	7.4E-03	3.0E-03
C_0030	Betaine	C00719	HMDB00043	3.2E-02	5.7E-03	3.7E-02	3.8E-03	3.5E-02	3.4E-03
C_0119	Butyrylcarnitine	C02862	HMDB02013	3.6E-04	3.3E-05	2.3E-04	7.6E-05	2.2E-04	3.3E-05
C_0081	Carnitine	C00318, C00487, C15025	HMDB00062	2.9E-02	3.0E-03	1.8E-02	4.7E-03	2.1E-02	2.7E-03
C_0116	Carnosine	C00386	HMDB00033	1.9E-04	4.6E-05	1.9E-04	5.0E-05	3.3E-04	1.3E-04
A_0102	Cholic acid	C00695	HMDB00619	2.9E-04	6.8E-05	5.4E-04	8.1E-04	2.1E-04	2.4E-05
C_0020	Choline	C00114	HMDB00097	2.3E-02	2.0E-03	2.1E-02	2.8E-03	1.7E-02	2.7E-03
A_0050	*cis* -Aconitic acid	C00417	HMDB00072	1.9E-03	1.0E-04	3.9E-03	5.9E-04	3.1E-03	3.0E-04
A_0062	Citric acid	C00158	HMDB00094	3.3E-02	3.9E-03	7.9E-02	1.8E-02	5.9E-02	8.9E-03
C_0091	Citrulline	C00327	HMDB00904	1.5E-02	1.9E-03	4.5E-02	1.3E-02	3.1E-02	3.4E-03
C_0045	Creatine	C00300	HMDB00064	1.2E-01	2.0E-02	6.9E-02	2.2E-02	6.7E-02	2.4E-02
C_0027	Creatinine	C00791	HMDB00562	8.3E-03	6.9E-04	3.2E-02	1.7E-02	1.9E-02	3.6E-03
C_0014	Cyclohexylamine	C00571		1.2E-04	N.A.	1.9E-04	7.2E-05	9.1E-05	N.A.
C_0115	Cystathionine	C00542, C02291	HMDB00099	4.2E-04	1.3E-04	1.0E-03	2.4E-04	7.4E-04	1.7E-04
C_0149	Cysteine glutathione disulfide	C05526	HMDB00656	3.5E-03	7.8E-04	4.2E-03	1.0E-03	3.1E-03	4.3E-04
C_0123	Cystine	C00491, C01420	HMDB00192	7.7E-03	6.4E-04	6.0E-03	8.0E-04	6.3E-03	5.2E-04
C_0127	Cytidine	C00475	HMDB00089	3.1E-03	2.0E-04	3.4E-03	3.3E-04	3.0E-03	2.3E-04
C_0024	Cytosine	C00380	HMDB00630	N.A.	N.A.	1.0E-04	9.2E-05	3.9E-05	1.5E-05
A_0047	Decanoic acid	C01571	HMDB00511	5.6E-04	8.2E-05	3.9E-04	1.9E-05	4.2E-04	1.2E-04
C_0022	Diethanolamine	C06772	HMDB04437	4.8E-04	2.1E-04	7.3E-04	5.4E-04	4.3E-04	1.9E-04
C_0131	Dyphylline	C07819		7.6E-03	1.6E-03	5.9E-03	2.0E-03	7.4E-03	7.1E-04
C_0061	Ectoine	C06231		N.A.	N.A.	4.6E-04	2.3E-04	2.6E-04	2.2E-05
A_0067	Erythrose 4-phosphate	C00279, C03604	HMDB01321	N.A.	N.A.	6.6E-05	N.A.	1.4E-04	4.8E-05
C_0002	Ethanolamine	C00189	HMDB00149	3.5E-03	4.4E-04	3.2E-03	3.1E-04	2.7E-03	2.7E-04
A_0029	Ethanolamine phosphate	C00346	HMDB00224	8.8E-04	2.4E-04	8.4E-04	1.4E-04	6.9E-04	1.4E-04
[illegible]	[illegible]		[illegible]	[illegible]	[illegible]	[illegible]	[illegible]	[illegible]	[illegible]

A_0081	Fructose 6-phosphate	C05345, C00085	HMDB00124	1.2E-04	N.A.	N.A.	N.A.	N.A.	N.A.
A_0013	Fumaric acid	C00122	HMDB00134	5.8E-04	1.8E-04	8.1E-04	2.7E-04	5.9E-04	8.8E-05
C_0016	GABA	C00334	HMDB00112	1.6E-04	N.A.	2.1E-04	4.6E-05	1.7E-04	2.0E-05
C_0094	Galactosamine<br>Glucosamine	C02262, C00329	HMDB01514	N.A.	N.A.	7.1E-05	N.A.	9.4E-05	1.0E-05
C_0067	Gln	C00064, C00303, C00819	HMDB00641, HMDB03423	1.2E-01	1.9E-02	1.3E-01	1.5E-02	1.4E-01	1.2E-02
C_0070	Glu	C00025, C00217 ,C00302	HMDB00148, HMDB03339	6.2E-02	3.3E-03	4.2E-02	1.1E-02	2.4E-02	6.6E-03
A_0066	Gluconic acid	C00257	HMDB00625	6.9E-04	8.6E-05	1.3E-03	4.6E-04	9.2E-04	1.8E-04
C_0093	Gluconolactone	C00198	HMDB00150	4.0E-04	4.8E-05	7.0E-04	2.7E-04	5.2E-04	9.4E-05
A_0080	Glucose 6-phosphate	C00668, C01172, C00092	HMDB01401	6.0E-04	1.7E-04	4.1E-04	1.1E-04	3.2E-04	1.1E-04
A_0065	Glucuronic acid<br>Galacturonic acid	C00191, C00333	HMDB00127, HMDB02545	2.4E-04	2.8E-05	7.6E-04	5.5E-04	4.3E-04	6.6E-05
A_0024	Glutaric acid	C00489	HMDB00661	1.6E-04	5.1E-06	2.6E-04	7.9E-05	2.3E-04	4.1E-05
C_0142	Glutathione (GSSG)_divalent	C00127	HMDB03337	1.9E-03	6.6E-04	3.0E-03	6.6E-04	2.1E-03	6.4E-04
C_0005	Gly	C00037	HMDB00123	3.3E-02	2.9E-03	4.7E-02	7.8E-03	4.1E-02	5.1E-03
C_0103	Gly-Asp			3.3E-04	6.7E-05	1.9E-04	1.0E-04	1.0E-04	3.4E-05
C_0048	Gly-Gly	C02037	HMDB11733	1.2E-04	N.A.	1.4E-04	N.A.	9.8E-05	N.A.
A_0011	Glyceric acid	C00258	HMDB00139, HMDB06372	1.1E-03	1.6E-04	1.0E-03	2.9E-04	9.0E-04	2.9E-04
C_0013	Glycerol	C00116	HMDB00131	6.7E-02	2.4E-02	6.5E-02	2.8E-02	7.2E-02	1.2E-02
A_0046	Glycerol 3-phosphate	C00093	HMDB00126	5.0E-04	1.1E-04	5.8E-04	1.1E-04	5.3E-04	1.0E-04
C_0132	Glycerophosphocholine	C00670	HMDB00086	6.1E-03	1.1E-03	4.4E-03	1.6E-03	2.3E-03	9.7E-04
A_0106	Glycocholic acid	C01921	HMDB00138	N.A.	N.A.	1.5E-04	4.6E-05	1.3E-04	N.A.
C_0090	Guanidinosuccinic acid	C03139	HMDB03157	3.1E-05	N.A.	5.8E-04	6.6E-04	1.8E-04	1.4E-04
A_0022	Heptanoic acid		HMDB00666	1.8E-04	2.4E-05	1.5E-04	2.7E-05	1.9E-04	5.9E-05
A_0015	Hexanoic acid	C01585	HMDB00535	3.8E-04	2.1E-05	3.6E-04	8.1E-05	3.8E-04	8.7E-05
A_0054	Hippuric acid	C01586	HMDB00714	3.2E-04	1.9E-04	1.2E-03	1.1E-03	5.3E-04	1.2E-04
C_0077	His	C00135, C00768, C06419	HMDB00177	2.3E-02	3.3E-03	2.2E-02	3.0E-03	2.1E-02	2.3E-03
C_0025	Histamine	C00388	HMDB00870	2.8E-04	8.6E-05	4.1E-04	6.5E-05	3.7E-04	7.7E-05
C_0124	Homocarnosine	C00884	HMDB00745	2.5E-04	7.3E-05	2.1E-04	5.5E-05	4.1E-04	2.2E-04
C_0102	Homocitrulline	C02427	HMDB00679	5.0E-04	5.3E-05	1.6E-03	3.9E-04	1.1E-03	2.0E-04
C_0033	Homoserine	C00263	HMDB00719	2.1E-04	1.9E-05	1.9E-04	3.3E-05	1.6E-04	4.7E-06
A_0055	Homovanillic acid	C05582	HMDB00118	2.1E-04	4.6E-05	5.4E-04	2.5E-04	3.2E-04	7.4E-05
C_0044	Hydroxyproline	C01157	HMDB00725	8.8E-03	6.0E-04	1.2E-02	1.8E-03	1.1E-02	1.6E-03
C_0023	Hypotaurine	C00519	HMDB00965	1.7E-03	2.3E-04	1.0E-03	1.9E-04	8.6E-04	1.7E-04
C_0046	Ile	C0040, C06418, C16434	HMDB00172	7.6E-02	1.3E-02	6.9E-02	1.2E-02	5.8E-02	7.5E-03
C_0041	Imidazole-4-acetic acid	C02835	HMDB02024	2.4E-04	4.8E-05	4.0E-04	1.4E-04	3.4E-04	5.6E-05
C_0078	Imidazolelactic acid	C05568		N.A.	N.A.	1.3E-04	3.3E-05	7.6E-05	1.2E-05
A_0019	Isethionic acid	C05123	HMDB03903	1.1E-03	2.2E-04	2.1E-03	7.8E-04	1.7E-03	4.7E-04
A_0004	Isobutyric acid<br>Butyric acid	C02632, C00246	HMDB01873, HMDB00039	1.7E-04	2.2E-05	1.7E-04	N.A.	1.7E-04	5.0E-05
A_0061	Isocitric acid	C00311	HMDB00193	2.8E-03	2.7E-04	5.7E-03	1.3E-03	4.4E-03	6.8E-04
C_0071	Isoglutamic acid	C05574		N.A.	N.A.	7.4E-05	N.A.	N.A.	N.A.
A_0007	Isovaleric acid<br>Valeric acid	C08262, C00803	HMDB00718, HMDB00892	3.2E-04	4.2E-05	3.0E-04	1.8E-05	3.3E-04	3.0E-05
A_0048	Isovalerylalanine<br>*N*-Acetylleucine	C02710	HMDB00747, HMDB11756	1.5E-04	N.A.	2.9E-04	1.8E-04	2.3E-04	5.7E-05
C_0129	Isovalerylcarnitine		HMDB00688	4.0E-04	3.7E-05	2.5E-04	5.5E-05	2.4E-04	3.3E-05
C_0110	Kynurenine	C00328, C01718	HMDB00684	1.5E-03	4.8E-04	2.0E-03	6.2E-04	1.8E-03	1.9E-04
A_0005	Lactic acid	C00186, C00256, C01432	HMDB00190, HMDB01311	8.2E-01	3.5E-01	7.8E-01	1.4E-01	5.9E-01	1.6E-01
A_0068	Lauric acid	C02679	HMDB00638	1.0E-03	1.1E-04	8.8E-04	1.7E-04	8.7E-04	1.2E-04
C_0047	Leu	C00123, C01570, C16439	HMDB00687	1.1E-01	2.4E-02	9.2E-02	8.1E-03	8.7E-02	1.1E-02
C_0068	Lys	C00047, C00739, C16440	HMDB00182, HMDB03405	1.1E-01	1.1E-02	1.1E-01	1.4E-02	1.0E-01	1.1E-02
A_0028	*m*-Hydroxybenzoic acid	C00587	HMDB02466	N.A.	N.A.	1.1E-04	4.2E-05	1.5E-04	N.A.
A_0026	Malic acid	C00149, C00497, C00711	HMDB00156, HMDB00744	8.2E-03	3.8E-03	1.5E-02	3.8E-03	9.6E-03	2.9E-03
C_0072	Met	C00073, C00855, C01733	HMDB00696	1.6E-02	1.5E-03	1.6E-02	2.7E-03	1.3E-02	1.7E-03
C_0083	Methionine sulfoxide	C02989	HMDB02005	3.8E-04	9.6E-05	5.4E-04	1.6E-04	5.1E-04	1.1E-04
C_0004	Methylguanidine	C02294	HMDB01522	N.A.	N.A.	1.4E-03	N.A.	N.A.	N.A.
C_0008	Morpholine	C14452	HMDB31581	1.5E-04	N.A.	1.7E-04	6.1E-05	1.6E-04	7.9E-06
C_0028	Muscimol	C08311		N.A.	N.A.	1.4E-04	N.A.	N.A.	N.A.
A_0073	Myristoleic acid	C08322	HMDB02000	1.2E-04	N.A.	N.A.	N.A.	N.A.	N.A.
C_0019	*N*,*N*-Dimethylglycine	C01026	HMDB00092	2.2E-03	3.4E-04	5.6E-03	8.6E-04	4.0E-03	6.9E-04
A_0023	*N*-Acetyl-β-alanine	C01073		N.A.	N.A.	1.6E-04	6.7E-05	8.2E-05	N.A.
A_0051	*N*-Acetylaspartic acid	C01042	HMDB00812	N.A.	N.A.	3.3E-04	1.5E-04	2.2E-04	5.0E-05
C_0114	N-Acetylgalactosamine-1<br>N-Acetylmannosamine-1<br>N-Acetylglucosamine-1	C01132, C00645, C00140	HMDB00853, HMDB01129, HMDB00215	3.0E-03	5.2E-04	2.6E-03	4.2E-04	2.8E-03	5.4E-04
C_0113	N-Acetylgalactosamine-2<br>N-Acetylmannosamine-2<br>N-Acetylglucosamine-2	C01132, C00645, C00140	HMDB00853, HMDB01129, HMDB00215	1.4E-03	2.8E-04	1.8E-03	3.6E-04	1.6E-03	2.2E-04
A_0086	*N*-Acetylglucosamine 6-phosphate	C00357	HMDB01062	N.A.	N.A.	1.2E-04	N.A.	N.A.	N.A.
A_0060	*N*-Acetylglutamine		HMDB06029	N.A.	N.A.	1.5E-04	4.1E-05	1.2E-04	2.1E-05
A_0016	*N*-Acetylglycine		HMDB00532	1.5E-04	3.6E-05	3.8E-04	5.5E-05	3.6E-04	4.0E-05
C_0104	*N*-Acetylhistidine	C02997		N.A.	N.A.	9.0E-05	6.2E-05	5.5E-05	1.6E-05
C_0098	*N*-Acetyllysine	C12989	HMDB00446	1.4E-04	N.A.	2.6E-04	9.4E-05	1.7E-04	3.3E-05
C_0069	*N*-Acetylserine		HMDB02931	N.A.	N.A.	2.5E-04	8.3E-05	1.4E-04	3.1E-05
C_0062	*N*-Ethylmaleimide_+H_2O	C02441		N.A.	N.A.	1.3E-04	5.6E-05	1.5E-04	N.A.
C_0075	N^1-Methyl-4-pyridone-5-carboxamide	C05843	HMDB04194	N.A.	N.A.	5.3E-04	N.A.	N.A.	N.A.
C_0088	N^5-Ethylglutamine	C01047		2.2E-03	4.1E-04	4.5E-03	1.2E-03	4.5E-03	7.2E-04
C_0101	N^6,N^6,N^6-Trimethyllysine	C03793	HMDB01325	9.8E-04	1.1E-04	2.7E-03	1.0E-03	1.8E-03	2.4E-04
C_0099	N^6-Acetyllysine	C02727	HMDB00206	2.0E-04	2.4E-05	3.2E-04	4.7E-05	2.8E-04	2.9E-05
C_0079	N^6-Methyllysine	C02728	HMDB02038	3.3E-04	6.2E-05	9.5E-04	2.6E-04	6.6E-04	1.1E-04
C_0097	N^8-Acetylspermidine	C01029	HMDB02189	8.7E-05	2.7E-05	1.9E-04	3.8E-05	1.2E-04	1.8E-05
C_0037	Nicotinamide	C00153	HMDB01406	1.2E-03	4.8E-05	9.4E-04	2.0E-04	8.5E-04	1.3E-04
C_0100	N_{ω}-Methylarginine			9.6E-05	3.0E-05	1.2E-04	2.0E-05	9.0E-05	1.7E-05

C_0080	*O* -Acetylhomoserine<br>2-Aminoadipic acid	C01077, C00956	HMDB00510	3.3E-04	6.0E-05	5.7E-04	8.8E-05	4.4E-04	6.8E-05
A_0030	Octanoic acid	C06423	HMDB00482	4.1E-04	1.1E-04	3.3E-04	2.8E-05	3.2E-04	3.9E-05
C_0138	Ophthalmic acid		HMDB05765	9.1E-05	3.1E-05	1.1E-04	3.3E-05	9.6E-05	1.8E-05
C_0050	Ornithine	C00077, C00515, C01602	HMDB00214, HMDB03374	1.2E-02	1.2E-03	1.5E-02	3.1E-03	1.1E-02	2.9E-03
A_0037	*p* -Anisic acid<br>*o* -Hydroxyphenylacetic acid<br>Mandelic acid<br>Phenoxyacetic acid	C02519, C05852, C01983<br>C01984<br>C02181	HMDB01101, HMDB00669, HMDB00703	1.5E-04	8.6E-06	1.9E-04	5.6E-05	1.6E-04	1.5E-05
A_0071	Pantothenic acid	C00864	HMDB00210	3.1E-04	7.0E-05	5.1E-04	2.7E-04	3.0E-04	9.5E-05
A_0038	Pelargonic acid	C01601	HMDB00847	4.7E-04	6.1E-05	4.4E-04	4.1E-05	4.2E-04	6.9E-05
C_0085	Phe	C00079, C02057, C02265	HMDB00159	3.7E-02	5.2E-03	3.7E-02	5.0E-03	3.2E-02	2.8E-03
A_0064	Phenaceturic acid	C05598	HMDB00821	8.3E-04	3.3E-04	4.3E-03	3.5E-03	2.7E-03	7.4E-04
A_0069	Phosphocreatine	C02305	HMDB01511	1.3E-04	N.A.	2.2E-04	7.8E-05	1.7E-04	4.8E-05
C_0096	Phosphorylcholine	C00588	HMDB01565	2.5E-03	6.0E-04	2.2E-03	5.2E-04	1.4E-03	5.1E-04
C_0043	Pipecolic acid	C00408	HMDB00070, HMDB00716, HMDB05960	2.1E-03	3.8E-04	5.8E-03	1.0E-03	4.5E-03	1.0E-03
C_0007	Piperidine	C01746		4.5E-04	1.4E-04	1.2E-03	8.6E-04	4.2E-04	2.6E-04
C_0029	Pro	C00148, C00763, C16435	HMDB00162 ,HMDB03411	5.5E-02	5.9E-03	6.0E-02	1.1E-02	5.0E-02	4.9E-03
C_0009	Putrescine	C00134	HMDB01414	2.3E-04	1.3E-05	2.5E-04	4.6E-05	2.2E-04	3.2E-05
C_0086	Pyridoxal	C00250	HMDB01545	1.5E-04	4.2E-05	1.4E-04	2.4E-05	1.2E-04	3.1E-05
A_0053	Pyrophosphate	C00013	HMDB00250	N.A.	N.A.	2.3E-03	N.A.	N.A.	N.A.
A_0003	Pyruvic acid	C00022	HMDB00243	1.1E-02	5.4E-03	1.5E-02	2.1E-03	1.3E-02	4.1E-03
A_0076	Ribulose 5-phosphate	C00199, C01101	HMDB00618	9.8E-05	N.A.	2.1E-04	1.0E-04	1.6E-04	5.0E-05
C_0147	*S* -Adenosylhomocysteine	C00021	HMDB00939	2.2E-05	2.6E-06	2.6E-05	5.3E-06	2.5E-05	3.3E-06
C_0148	*S* -Adenosylmethionine	C00019	HMDB01185	4.2E-05	1.0E-05	9.2E-05	2.1E-05	7.9E-05	6.2E-06
C_0052	*S* -Methylcysteine		HMDB02108	6.9E-04	5.3E-05	6.7E-04	9.4E-05	5.3E-04	7.0E-05
C_0010	Sarcosine	C00213	HMDB00271	5.1E-04	4.3E-05	1.1E-03	1.9E-04	7.6E-04	1.8E-04
C_0106	SDMA		HMDB03334	1.8E-04	2.3E-05	6.1E-04	2.0E-04	3.7E-04	7.9E-05
A_0085	Sedoheptulose 7-phosphate	C05382	HMDB01068	N.A.	N.A.	1.3E-04	2.7E-05	1.8E-04	7.0E-05
C_0021	Ser	C00065, C00716, C00740	HMDB00187, HMDB03406	4.4E-02	6.3E-03	3.7E-02	7.6E-03	3.1E-02	3.9E-03
C_0120	Ser-Glu			1.0E-04	2.5E-05	1.7E-04	1.0E-04	7.7E-05	3.7E-05
C_0092	Serotonin	C00780	HMDB00259	1.3E-03	3.3E-04	1.7E-03	3.3E-04	1.8E-03	2.1E-04
C_0066	Spermidine	C00315	HMDB01257	7.0E-04	1.9E-04	6.2E-04	1.1E-04	4.5E-04	1.4E-04
C_0063	Stachydrine	C10172	HMDB04827	9.4E-04	4.0E-04	3.8E-03	8.9E-04	5.3E-03	1.9E-03
A_0017	Succinic acid	C00042	HMDB00254	2.4E-03	4.8E-04	2.8E-03	8.5E-04	1.6E-03	3.4E-04
A_0083	Sulfotyrosine			N.A.	N.A.	1.5E-04	5.4E-05	1.3E-04	2.7E-05
A_0036	Tartaric acid	C00898	HMDB00956	1.1E-03	5.3E-04	2.4E-03	5.1E-04	2.4E-03	5.8E-04
C_0038	Taurine	C00245	HMDB00251	2.1E-02	2.8E-03	1.7E-02	4.4E-03	1.7E-02	3.8E-03
A_0113	Taurocholic acid	C05122	HMDB00036	1.1E-03	4.7E-04	1.6E-03	8.5E-04	2.0E-03	1.5E-03
A_0040	Terephthalic acid	C06337	HMDB02428	2.4E-04	2.8E-05	2.3E-04	4.8E-05	2.4E-04	3.7E-05
C_0130	Tetrahydrouridine			1.5E-04	N.A.	2.1E-04	2.4E-05	2.1E-04	1.2E-05
C_0133	Thiamine	C00378	HMDB00235	1.1E-04	4.1E-05	1.0E-04	2.0E-05	7.9E-05	2.5E-05
C_0146	Thiamine phosphate	C01081	HMDB02666	1.1E-04	1.2E-05	1.2E-04	1.3E-05	1.1E-04	1.6E-05
C_0032	Thr	C00188, C00820	HMDB00167	5.1E-02	8.1E-03	5.9E-02	1.4E-02	4.6E-02	6.1E-03
C_0121	Thr-Asp			6.7E-05	1.2E-05	1.2E-04	5.9E-05	7.1E-05	6.5E-05
A_0027	Threonic acid	C01620	HMDB00943	1.8E-03	3.1E-04	2.4E-03	8.5E-04	2.0E-03	2.6E-04
C_0126	Thymidine	C00214	HMDB00273	3.8E-04	6.0E-05	4.5E-04	1.0E-04	4.5E-04	4.5E-05
C_0073	Triethanolamine	C06771		6.1E-05	2.2E-05	5.6E-05	N.A.	5.5E-05	N.A.
C_0056	Trigonelline	C01004	HMDB00875	5.2E-04	9.2E-05	3.5E-03	2.0E-03	1.4E-03	8.6E-04
C_0006	Trimethylamine *N* -oxide	C01104	HMDB00925	1.9E-03	1.1E-03	7.5E-03	5.1E-03	4.1E-03	8.0E-04
C_0109	Trp	C00078, C00525, C00806	HMDB00929	3.7E-02	4.6E-03	2.1E-02	5.9E-03	2.5E-02	3.0E-03
C_0095	Tyr	C00082, C01536, C06420	HMDB00158	3.0E-02	3.5E-03	2.2E-02	3.4E-03	1.8E-02	1.5E-03
A_0059	Undecanoic acid		HMDB00947	2.2E-04	6.2E-05	1.7E-04	1.9E-05	2.1E-04	9.3E-05
C_0026	Uracil	C00106	HMDB00300	4.8E-04	6.1E-05	4.5E-04	8.5E-05	3.4E-04	3.1E-05
C_0001	Urea	C00086	HMDB00294	8.6E-03	3.7E-04	2.1E-02	4.7E-03	1.6E-02	2.1E-03
A_0043	Uric acid	C00366	HMDB00289	8.5E-03	1.0E-03	7.9E-03	7.9E-04	7.1E-03	9.0E-04
C_0128	Uridine	C00299	HMDB00296	9.7E-04	1.8E-04	2.7E-03	1.2E-03	1.8E-03	2.5E-04
C_0059	Urocanic acid	C00785	HMDB00301	9.6E-05	9.6E-06	9.6E-05	1.9E-05	9.4E-05	6.7E-06
C_0031	Val	C00183, C06417, C16436	HMDB00883	1.1E-01	1.6E-02	8.5E-02	1.0E-02	8.1E-02	7.2E-03
A_0012	XA0002			3.6E-04	9.5E-05	3.8E-04	1.2E-04	4.1E-04	9.8E-05
A_0031	XA0004 (2-Keto-glutaramic acid)		HMDB0001552	2.0E-04	3.4E-05	4.5E-04	2.6E-04	2.0E-04	6.9E-05
A_0041	XA0012			6.8E-04	2.6E-04	9.2E-04	3.1E-04	8.4E-04	2.6E-04
A_0049	XA0013			2.6E-03	1.1E-03	6.8E-03	6.8E-03	3.7E-03	2.7E-03
A_0063	XA0019			2.6E-04	4.2E-05	3.2E-04	1.0E-04	3.3E-04	7.5E-05
A_0074	XA0027			4.2E-04	1.3E-04	3.6E-04	4.0E-05	2.9E-04	6.3E-05
A_0077	XA0033			3.6E-04	8.9E-05	2.8E-04	7.1E-05	2.1E-04	4.6E-05
A_0078	XA0035			2.0E-03	2.5E-04	3.5E-03	1.4E-03	2.8E-03	4.3E-04
A_0079	XA0036			1.9E-04	3.3E-05	3.5E-04	1.1E-04	3.1E-04	6.9E-05
C_0003	XC0001			3.4E-05	N.A.	1.5E-04	9.4E-05	5.7E-05	1.2E-05
C_0042	XC0016			6.3E-03	3.3E-03	1.3E-02	4.5E-03	1.7E-02	5.7E-03
C_0111	XC0061			9.3E-04	1.4E-04	3.6E-04	1.5E-04	4.8E-04	1.4E-04
C_0112	XC0065			N.A.	N.A.	7.2E-05	3.0E-05	N.A.	N.A.
C_0140	XC0120			9.2E-05	8.9E-06	8.0E-05	1.9E-05	9.0E-05	1.1E-05
C_0144	XC0126			1.1E-04	3.1E-05	5.5E-05	1.4E-05	5.4E-05	9.5E-06
C_0145	XC0132 (Galactosylhydroxylysine)		HMDB0000600	1.6E-04	4.4E-05	4.8E-04	2.1E-04	2.7E-04	4.2E-05
C_0011	β-Ala	C00099	HMDB00056	1.5E-03	2.4E-04	1.3E-03	2.2E-04	1.2E-03	1.9E-04
C_0065	γ-Butyrobetaine	C01181	HMDB01161	1.3E-03	6.8E-05	7.6E-04	1.5E-04	9.0E-04	1.2E-04
C_0141	γ-Glu-Val-Gly			N.A.	N.A.	7.4E-05	7.1E-06	7.4E-05	6.6E-06

C: cation mode, A: anion mode
