# Supplementary material for: Renoprotective effects of paramylon, a β-1,3-D-Glucan isolated from Euglena gracilis Z in a rodent model of chronic kidney disease
Source: PLoS One. 2020 Aug 7;15(8):e0237086. doi: 10.1371/journal.pone.0237086 (PMC7413521; doi:10.1371/journal.pone.0237086)
Supplement: Attachment — Submitted filename: Response to Reviewers.docx [file pone.0237086.s015.docx]

PLOS ONE Manuscript ID: PONE-D-19-35917

COMMENTS FOR THE REVIEWERS:

We are grateful to reviewer #1 for the critical comments and useful suggestions that have helped us to improve our paper. As indicated in the responses that follow, we have taken all these comments and suggestions into account in the revised version of our paper. **Please note: all page directions apply to the manuscript in red ink version ‘Revised Manuscript with Track Changes’ (length 59 pages)**

Before the response of point by point, we should tell you the following. In the first submission, we used t-test in Figure 2C, Figure 3A-E, and Figure 4. In the revised manuscript, we used ANOVA with Tukey’s post hoc test for multiple comparisons in those figures. As a result, statistical difference of some data became smaller and we corrected the description of the abstract, results, and discussion section.

Reviewer #1: The manuscript submitted by Nagayama et al describes the renoprotective effects of paromylon, a Beta-1,3-D-Glucan in a rodent model of chronic kidney disease. The treatment showed improvement of the kidney function and decreased accumulation of TCA cycle metabolites and uremic toxins.

Major comments:
-Figure 2 a, b and C: use arrows to show where the injury/damage is on the pictures for glomerulosclerosis, tubular injury, glomerular hypertrophy.

**We showed the sites of glomerulosclerosis or tubular injury with arrows in new Fig 1C and D. We believe glomerular area is obvious without arrows in new Fig 1E.**

- Improve the images quality. Show the bar graphs with dots for each mouse.
 **We improved the images quality and made the bar graphs with dots for each mouse in new Fig 1C -F.**

- Figure 1 can be joint to Figure 2, as well as the results sections linked to both figures.
 **We jointed Figure 1 and Figure 2, as well as the results sections linked to both figures.** **Because of this, we renumbered all the figures.**

-Figure 3: Improve the images quality. Use arrows to show where the injury/damage is.

**We improved the images quality and showed the fibrotic area, the α-SMA positive area, or the sites of ED-1, CD3, or PCNA positive cells with arrows in new Fig 2A-E.**

Don't use the word tendency to describe the non-significant differences in the results section.

**We quitted the word tendency to describe the non-significant differences in the results section. (page 16, line 278, page 19, line 332, page 20, line 337)**

Use dots to represent each mouse on the graphs (dot plot).
**We** **made the bar graphs with dots for each mouse in new Fig 2A-E.**

- Figure 4: It would be nice to see MMP9, IL10 and ROS levels in the kidneys too. It would be interesting to evaluate if there is modulation of the macrophage profile induced by paromylon.
 **We investigated the renal gene levels of matrix metalloproteinase 9 (MMP9), inducible nitric oxide synthase 2 (NOS2), and interferon-γ(IFN-γ) as** **proinflammatory M1 type macrophage marker, and IL-10 and IL-4 as anti-inflammatory M2 type macrophage marker. (page 20, line 340-346)**

**There were no differences of the gene expressions of *Mmp9*, *Ifng*, *Nos2*, *Il-10*, and *Il-4* between the Nx group and the Nx + PAR group. (new Figs 3G-K)**

-Figure 5: The treatment decreases the accumulation of TCA cycle metabolites. Improve the discussion about these data. Correlate to improvement of inflammatory parameters such as IL1b.

**We discussed the accumulation of TCA cycle metabolites and the improvement by paramylon. (page 33, line 541-548)**

Improve the quality of the graphs. An alternative to better show this data is to build a heatmap.

**We improved the images quality and made a heatmap as a new Fig 4A.**

The results section “Identification of some uremic toxins using CE-TOFMS analysis” needs to be improved. The title could be changed to something like: Accumulation of TCA cycle metabolites and uremic toxins are decreased in the serum by paromylon treatment.

**We changed the title as you suggested. (page 22, line 377-379)**

This section should be better described because these results fall from the sky, without any introduction about why to look at this (TCA cycle etc).
 **We investigated** **uremic solutes that indicated the effects of paramylon and identified six anionic and 21 cationic compounds. To be honest, we did not know the characteristics of these compounds. Because you kindly taught us that TCA cycle-related metabolites were contained, we corrected the results and discussion of this section. (page 24, line 415-419, page 33, line 541-548)**

Figure 6: Since Beta glucans are found in fungi species, would you consider changes in fungi species in the microbiome induced by the treatment?

**We reanalyzed the gut microbiome with next-generation DNA sequencing. We did not get PCR products of fungi species, although we got PCR products of bacterial species. Therefore, we could not evaluate the changes in fungi species in the microbiome induced by the treatment.**

Minor Comments:

-The word “tendency” should be replaced.

**We quitted the word “tendency” in the results section. (page 16, line 278, page 19, line 332, page 20, line 337)**

-Improve the quality of all images and include the scale bar.

**We improved the quality of all images and included the scale bar on** **all the renal histology images.**

We are grateful to reviewer #2 for the critical comments and useful suggestions that have helped us to improve our paper. As indicated in the responses that follow, we have taken all these comments and suggestions into account in the revised version of our paper. **Please note: all page directions apply to the manuscript in red ink version ‘Revised Manuscript with Track Changes’ (length 59 pages)**

Before the response of point by point, we should tell you the following. In the first submission, we used t-test in Figure 2C, Figure 3A-E, and Figure 4. In the revised manuscript, we used ANOVA with Tukey’s post hoc test for multiple comparisons in those figures. As a result, statistical difference of some data became smaller and we corrected the description of the abstract, results, and discussion section.

Reviewer #2: This is an interesting manuscript which authors indicating paramylon reduces renal tissue damage and inflammation, however, paramylon has no effect on the intestinal microbiota. However, several points to be revised in the present manuscript to be accept in Plos One, described as follow.
Introduction:
- Must be revised and include an information about the impact of altered intestinal microbiota on CKD progression and the beneficial of dietary fibers in this context.

**We included references about the impact of altered intestinal microbiota and the beneficial of dietary fibers on CKD progression and revised the Introduction. (page 5, line 76-79)**

- Must be cited the original studies about the biological effects of dietary B-glucans described in lines 58-60.

**We cited the original studies about the biological effects of dietary B-glucans. (page 4, line 59-61)**

- After citing once “Euglena gracilis Z”, abreviate as “E. gracilis Z”, as described in the others manuscript’s sections.

**We abbreviated “Euglena gracilis Z”as “E. gracilis Z”. (page 4, line 61)**

- Instead of use “CKD rats”, substitute for “CKD rat model”.

**We substituted “CKD rats” for “CKD rat model”.**

Results:
- Revised the use of the “on the other hand” in description of results.

**We deleted “on the other hand” in description of results. (page 16, line 279, page 20, line 333, page 23, line 392, page 24, line 412)**

- The scale bars must be insert in the images.

**We inserted the scale bars on the all the renal histology images.**

- In the figure 4, identify de graphics with letters (A-F), the control group was not demonstrated in this figure.

**We added the data of the control group in the new figure 3 with letters (A-K)**

- Attenuate the phrase described in lines 306-310. The authors just analyzed gene expression.
 **We attenuated the phrase described in lines 306-310. (page 20, line 337-339)**

- The authors reported no differences in bacteria genera or clusters in microbiota. Maybe the differences could be observed among the species, instead of genera or clusters. Metagenomic assay will be very much appreciated to verify this point.

**We reanalyzed the gut microbiome due to next-generation DNA sequencing which was higher sensitive compared with T-RFLP analysis. As a result, there were significant differences of distribution of some genera between the CKD group and the CKD treated group. We corrected the methods, the results, and the discussion section.**

- In the literature has been hypothesized that increased gut permeability contributes to inflammation in patients with advanced CKD. Was the gut tissue analyzed in the present study? Sometimes the amelioration induced by paramylon could be related to improve de mucosal permeability, and it could be discussed.
 **We agree with you in that** **paramylon could be related to improve the gut permeability. Unfortunately, we did not get the gut tissue to analyze. Thus, we discussed the possibility that paramylon was related to improve the gut permeability. (page 35, line 574-577)**

Discussion

-The results are very poor discussed. The authors described results in this section instead of discuss them. The discussion must be revised and intersect the findings with data from literature.
 **We revised the discussion section.**

-The limitations of techniques used to characterize intestinal microbiome must be discussed. The interface between gut microbiota and CKD must be explored.

**We reanalyzed the gut microbiome due to next-generation DNA sequencing which was higher sensitive compared with T-RFLP analysis. We discussed the interface between gut microbiota and CKD including our data. (page 33, line 552-page 34, line 564)**

In line 452 - “It is unknown whether 5-hydroxylysine, argininosuccinic acid,
N6-methyllysine, N8-acetylspermidine, O-acetylhomoserine, and
galactosylhydroxylysine are uremic toxins”. Were these compounds described in CKD patients or another rodent model of CKD? What the relevance these compounds whether they are unknown their classification as uremic toxins?

**As far as we know, there were no previous reports that these compounds were accumulated in CKD patients or CKD animal models. We summarized previous reports as uremic toxin of six anionic and 21 cationic compounds we identified in the present study in new Table 2.**
