# Supplementary material for: Renoprotective effects of paramylon, a β-1,3-D-Glucan isolated from Euglena gracilis Z in a rodent model of chronic kidney disease
Source: PLoS One. 2020 Aug 7;15(8):e0237086. doi: 10.1371/journal.pone.0237086 (PMC7413521; doi:10.1371/journal.pone.0237086)
Supplement: Attachment — Submitted filename: Response to Reviewers.docx [file pone.0237086.s016.docx]

PLOS ONE Manuscript ID: PONE-D-19-35917R1

COMMENTS FOR THE REVIEWERS:

We are grateful to reviewer #1 for the critical comments and useful suggestions that have helped us to improve our paper. As indicated in the responses that follow, we have taken all these comments and suggestions into account in the revised version of our paper. **Please note: all page directions apply to the manuscript in red ink version ‘Revised Manuscript with Track Changes’ (length 48 pages)**

Reviewer #1: The authors provided answer for all my points. The quality of this manuscript improved considerably, however in the results section, the results could be more explored and the authors could provide more background on why they performed those experiments, before presenting the results. **We added background on why we performed those experiments and interpretation of the results in the results section. (page 15, line 263-264, page 16, line 274, page 18, line 298-301, page 19, line 316-318, page 20, line 349-350, page 21, line 351- 353, page 24, line 412-415, page 25, line 434-435)**

We are grateful to reviewer #2 for the critical comments and useful suggestions that have helped us to improve our paper. As indicated in the responses that follow, we have taken all these comments and suggestions into account in the revised version of our paper. **Please note: all page directions apply to the manuscript in red ink version ‘Revised Manuscript with Track Changes’ (length 48 pages)**

Reviewer #2: The findings reported by Nagayama and colleagues are interesting and present potential to be published in Plos One. Although, the authors had accepted all suggestions made by reviewers in the first moment, the article still need to be adequate to formal language, and the revision by a native English speaker is mandatory. In this second revision, the discussion section needs more attention, the authors use inadequate grammar and unnecessary sentences to justify the findings, some examples are listed below.

Discussion
- - Table 2 is suggested as supplemental data;

**We replaced Table 2 with supplemental Table 7. (page 27, line 476, page 30, line 490) Because of this, we rearranged references.**

- - The sentences need to be better connected, using adequate English grammar

**We submitted our manuscript to a native English speaker and we corrected the manuscript.**

- - In line 507, “we investigated whether if paramylon inhibited absorption of uremic toxin with CE-TOFMS analysis”. Review the use of whether/if

**We deleted ‘if’. (page 27, line 472)**

- - Some phrases depreciated this work and must be attenuated or excluded: “Unfortunately, we did not get the gut tissue to analyze”; There were several limitations in our study.

**We deleted the following descriptions; ‘In the literature it has been hypothesized that increased gut permeability contributes to inflammation in the advanced CKD [21]. The anti-inflammatory effect of paramylon could be related to improve the gut permeability. Unfortunately, we did not get the gut tissue to analyze.’ (page 31, line 511-514)**

**We deleted the following sentence; ‘There were several limitations in our study.’ (page 32, line 520) and replaced it to the attenuated phrases.**

- - Dear authors, the discussion must be carefully revised and interconnect the results and then submitted to a native English speaker.

**We carefully revised the discussion section interconnected with the results and we submitted our manuscript to a native English speaker.**
